# Transcriptome Analysis Reveals a Comprehensive Virus Resistance Response Mechanism in Pecan Infected by a Novel Badnavirus Pecan Virus

**DOI:** 10.3390/ijms232113576

**Published:** 2022-11-05

**Authors:** Jiyu Zhang, Tao Wang, Zhanhui Jia, Xiaodong Jia, Yongzhi Liu, Jiping Xuan, Gang Wang, Fan Zhang

**Affiliations:** Jiangsu Key Laboratory for the Research and Utilization of Plant Resources, Institute of Botany, Jiangsu Province and Chinese Academy of Sciences, Nanjing 210014, China

**Keywords:** pecan mosaic virus, fatty acids, cytokinin biosynthesis, ribosome, carbohydrate partitioning, leave chlorosis

## Abstract

Pecan leaf-variegated plant, which was infected with a novel badnavirus named pecan mosaic virus (PMV) detected by small RNA deep sequencing, is a vital model plant for studying the molecular mechanism of retaining green or chlorosis of virus-infected leaves. In this report, PMV infection in pecan leaves induced PAMP-triggered immunity (PTI) and effector-triggered immunity (ETI). PMV infection suppressed the expressions of key genes of fatty acid, oleic acid (C18:1), and very-long-chain fatty acids (VLCFA) biosynthesis, indicating that fatty acids-derived signaling was one of the important defense pathways in response to PMV infection in pecan. PMV infection in pecans enhanced the expressions of *pathogenesis*-*related protein* 1 (*PR*1). However, the transcripts of *phenylalanine ammonia*-*lyase* (*PAL*) and *isochorismate synthase* (*ICS*) were downregulated, indicating that salicylic acid (SA) biosynthesis was blocked in pecan infected with PMV. Meanwhile, disruption of auxin signaling affected the activation of the jasmonic acid (JA) pathway. Thus, C18:1 and JA signals are involved in response to PMV infection in pecan. In PMV-infected yellow leaves, damaged chloroplast structure and activation of mitogen-activated protein kinase 3 (MPK3) inhibited photosynthesis. Cytokinin and SA biosynthesis was blocked, leading to plants losing immune responses and systemic acquired resistance (SAR). The repression of photosynthesis and the induction of sink metabolism in the infected tissue led to dramatic changes in carbohydrate partitioning. On the contrary, the green leaves of PMV infection in pecan plants had whole cell tissue structure and chloroplast clustering, establishing a strong antiviral immunity system. Cytokinin biosynthesis and signaling transductions were remarkably strengthened, activating plant immune responses. Meanwhile, cytokinin accumulation in green leaves induced partial SA biosynthesis and gained comparatively higher SAR compared to that of yellow leaves. Disturbance of the ribosome biogenesis might enhance the resistance to PMV infection in pecan and lead to leaves staying green.

## 1. Introduction

Plants are constantly attacked by microbial pathogens. They have evolved intricate deference systems during the past 350 million years [1], including the physical barrier (thickness and trichomes), toxic compounds, secondary metabolites, and constitutive defense systems. Plants have evolved two-layered innate immune systems to defened against invading pathogens. Plants rely on pathogen-associated molecular patterns (PAMPs) to recognize pathogens by plant pattern recognition receptors (PRRs) and induce a basal-level resistance known as PAMP-triggered immunity (PTI) [2,3]. The other line of plant defense is stimulated by plant resistance (R) protein-mediated detection of pathogenic effectors, referred to as effector-triggered immunity (ETI), which is often associated with hypersensitive response (HR), a form of programmed cell death [2,4].

Viral pathogens are the major constraining factors for crop growth and production worldwide [5]. Plant viruses could efficiently utilize host resources for propagation and transform the host cell into a ‘Trojan horse’, sheltering enemies inside [6]. For most plant viruses, attachment to the chloroplast membrane is a signature infection pattern [7]. In fact, damage to the chloroplast for the virus was one of the pivotal steps in successful infection. Chloroplasts, which play a critical role in plant immunity in response to virus pathogens infection, could serve as sensors for detecting perturbations in the subcellular environment and actively communicate these signals from the chloroplast to the nucleus [8,9].

The plant defense system is regulated by numerous phytohormones, such as salicylic acid (SA), jasmonic acid (JA), abscisic acid (ABA), ethylene (ET), gibberellin (GA), brassinosteroids (BR), and cytokinin (CK) [1,10,11]. Plant immunity is upregulated by cytokinin through the elevation of SA-dependent defense responses; however, in SA, feedback inhibited cytokinin signaling [11]. Upon viral infection, auxin signaling is required to activate the JA pathway, and auxin positively interacts with the JA pathway against necrotrophic pathogens [12]. Suppressing the expression of *GhSSI2s* reduces the oleic acid (C18:1) level and enhanced cotton Verticillium wilt and Fusarium wilt resistance. Knockdown of *GhSSI2s* triggers a lesion mimic phenotype with an elevated SA level but without activating the JA signaling pathway [13]. A reduced C18:1 level could modulate defense signaling by either directly altering the expression of various defense-associated genes or affecting the activities of the encoded proteins. Thus, C18:1 functions as a signaling mediator in plant resistance against several pathogens.

**Figure 1:**
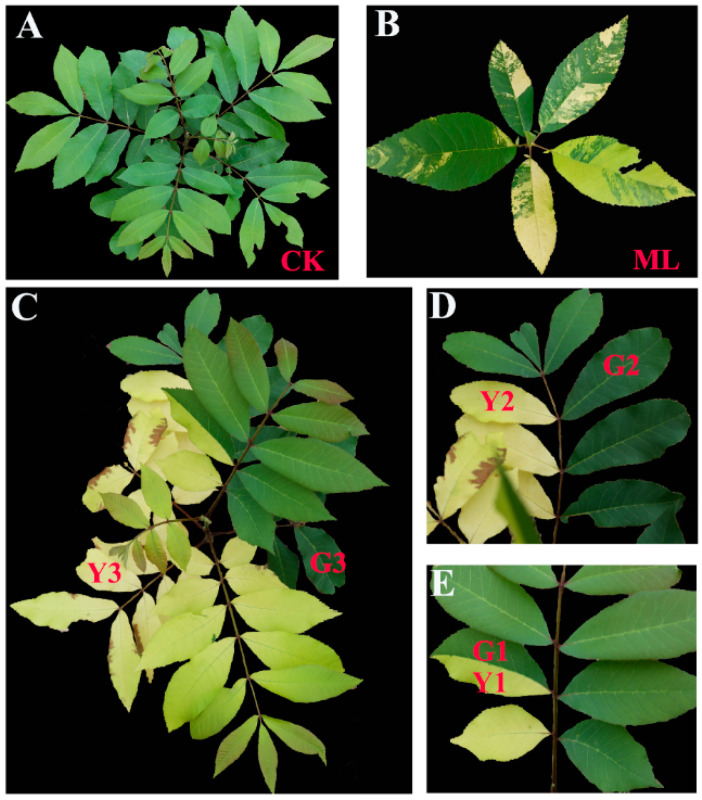
The symptoms of pecan plants infection with PMV. The early developmental stage of annual seedlings bore yellow margins and green interior leaves (**B**). The leaf-variegated plant bore half green leaves and half yellow leaves when developing a pinnately compound leaf (**C**–**E**). The mosaic leaves (ML, (**B**)) were photographed at June 2019. The green leaves (G1, G2, G3) and yellow leaves (Y1, Y2, Y3) of PMV-infected plants and the leaves of healthy plants (**A**) were photographed in June 2020.

A naturally occurring leaf-variegated pecan plant was obtained from millions of six-month-old pecan seedlings from Nanjing, Jiangsu province in China. Early one-year leaf-variegated plants bore yellow margins and green interior leaves (Mosaic leaf, ML, Figure 1B). It was most surprising and interesting that the leaf-variegated plant, when developing a pinnately compound leaf, had half green leaves and half yellow leaves (Figure 1C), and the pinnately compound leaf had half small green leaves and half small yellow leaves (Figure 1D), small leaf had half a piece of yellow (Figure 1E). In contrast, the typical normal seedling exhibited dark green-colored leaves (Figure 1A). So, it was an ideal material for studying the mechanism of virus invasion plants. In order to prove that the symptom was reproducible, we grafted leaf-variegated pecan plant branches onto normal pecan seedlings through spring branch grafting in 2022. The result showed that the new leaves from the same grafted branches showed the same symptom as the original leaf-variegated pecan plant (Figure S1). Deep sequencing and assembly of virus-derived small interfering RNAs has proven to be a highly efficient approach for virus discovery [14]. In this study, deep sequencing and assembly of virus-derived small interfering RNAs were performed to accurately detect viruses in pecans. Small green leaves (G1, G2, and G3) and small yellow leaves (Y1, Y2, and Y3) of infected PMV pinnate leaves and mosaic leaves (ML) were harvested, and their global transcriptional profiles were assessed by means of RNA-seq. Candidate genes with potential involvement in driving plant defense systems were identified. Why small PMV-infected leaves remained green or chlorosis in pecan pinnate leaves was investigated in our study.

## 2. Results

### 2.1. Virus Discovery by Deep Sequencing and Assembly of Virus-Derived Small Silencing RNAs

Deep sequencing and assembly of virus-derived small interfering RNAs was performed to find out whether it was caused by virus infection in variegated-leaves plants according to the workflow of VirusDetect [14]. After trimming the adaptor sequences and filtering for transfer and ribosomal RNAs, 2.37 × 10^7^ reads were obtained. The percentages of 16 to 34 nt siRNAs identified are shown in Figure S2. The 21 nt sRNAs predominated and accounted for almost 40% of the total sRNAs. A total of 22 nt and 24 nt sRNAs accounted for 11.83% and 16.04%, respectively. Our results were consistent with former reports that 21 nt, 22 nt, and 24 nt sRNAs were predominant in viroid-infected plants [15]. Single-base resolution maps of all redundant sRNAs, along with the genomes, were created using bowtie tools and in-house Perl scripts [16]. Then, the unmapped sRNAs were assembled using the Velvet (version 1.2.10) [17] and the results were shown as Tables S1 and S2. Assembled contigs were annotated to host genome, NCBI non-redundant protein sequences (NCBI Nr), NCBI non-redundant nucleotide sequences (NCBI Nt), GenBank virus nucleotide reference sequences, GenBank virus protein reference sequences. Virus-related contigs annotation information contained virus nucleotide reference sequences annotated, or virus protein reference sequences annotated but not by host genome and virus nucleotide reference sequences annotated, and viruses only annotated by NCBI Nr/Nt (Table S3). To evaluate the effectiveness of the virus, the sRNAs, which were not matched to the host genome, were compared to nucleic acid sequences found in the virus reference sequences nucleotide database by assembly contigs blast (Table S4). The results showed that Rubus yellow net virus, Gooseberry vein banding virus, Taro bacilliform CH virus, and Pagoda yellow mosaic-associated virus were related to plants. Previous reports showed that Rubus yellow net virus [18], Gooseberry vein banding-associated virus [19], Taro bacilliform CH virus [20], and Pagoda yellow mosaic-associated virus [21] belong to Badnavirus, which could cause yellow mosaic symptoms in leaves. Taken together, our results showed that a novel badnavirus was discovered from pecan seedlings showing yellow mosaic symptoms (Figure 1), and the virus was tentatively named pecan mosaic virus (PMV).

### 2.2. Leaf Cross-Section Microstructure Analysis

**Figure 2:**
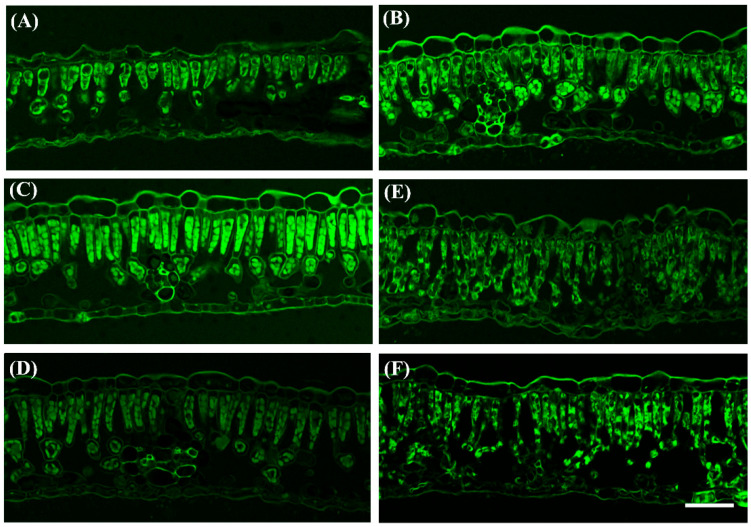
Leaf cross-section structures analysis in healthy plant leaves (CK, (**A**)), the mosaic leaves (**B**), the green leaves G1(**C**) and G2 (**D**), and yellow leaves Y1 (**E**) and Y2 (**F**) of PMV-infected plants. Bars = 20 μm.

Chloroplasts play vital roles in plant defense against microbial plant pathogens [6]. Chloroplasts are a prime target for viruses, and damage to the chloroplasts is one of the pivotal steps for viruses’ successful infection. Thus, leaf cross-section structures in healthy plant leaves (CK), the mosaic leaf (ML), the green leaves (G1 and G2), and yellow leaves (Y1 and Y2) of PMV-infected plants were studied in our study. Compared with CK, the palisade tissue of ML is arranged scattered, while the palisade tissue is arranged orderly and chloroplasts clustering (Figure 2 and Figure S3, ML). The palisade tissue and palisade tissue are arranged orderly, and chloroplast clustering was obvious in the green leaves (Figure 2 and Figure S3, G1 and G2) of the PMV-infected pecan plant. However, the palisade tissue and palisade tissues in yellow leaves were severely destroyed in PMV-infected pecan plants (Figure 2 and Figure S3, Y1 and Y2). These results showed that chloroplasts structures were severely damaged in the yellow leaves of PMV-infected pecan plants, but half the leaves of PMV-infected pecans stayed green (Figure 1) and triggered chloroplast clustering (Figure 2 and Figure S3), suggesting that the green leaves of PMV-infected pecan plants could establish a strong antiviral immunity system.

### 2.3. Characterization of the PMV-Infected Pecan Transcriptome

RNA-seq analysis was performed to provide a comprehensive profile of pecans in response to PMV in our study. Green (G1, G2, and G3) and yellow (Y1, Y2, and Y3) leaves in different positions of pinnately compound leaves, and mosaic leaves (ML) were harvested (Figure 1) for RNA sequencing. Illumina platform generated 486,954,046 raw reads. After filtering, 464,616,564 clean reads containing a total of 69.43 Gb clean nucleotides were obtained through stringent quality assessment and data filtering (Table S5). The clean reads were mapped to the pecan genome using the HISAT2 tool, and the total mapped reads, uniquely mapped reads and multiply mapped reads were summarized in Table S5. StringTie was applied to transcript assembly based on the read alignments. A total of 33,050 genes were identified with 1978 new genes after optimal gene structure prediction and alternative splicing analysis (Tables S6 and S7).

### 2.4. Global Transcriptome Changes in Pecan Infected with PMV

**Figure 3:**
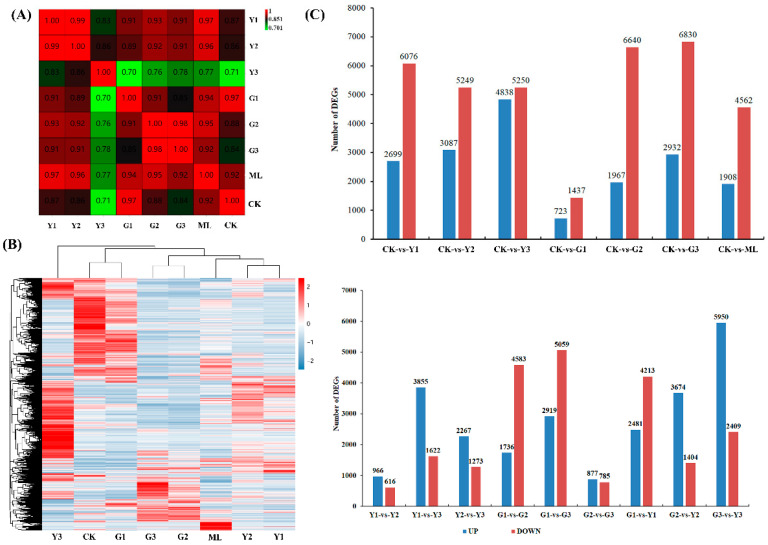
The principal component analysis between the two samples was calculated based on FPKM (**A**); (**B**): heatmap of RNA-seq transcriptome analysis for DEGs. (**C**): number of DEGs between two samples.

A principal component analysis (PCA) was performed with normalized read counts obtained from DESeq based on the prcomp function in the R environment (Figure 3A). The results showed that Y1 and Y2 had the highest correlation coefficient (0.99), followed by G2 and G3 (0.98) and G1 and CK (0.97). A total of 16,333 differentially expressed genes (DEGs) were identified in PMV-infected samples compared to noninfected plants (CK) (Table S8). To visualize the expression patterns of these DEGs at different samples, a heatmap was constructed on the basis of the fragments per kilobase of transcript per million mapped values (Figure 3B). DEGs with similar expression patterns were grouped, and the heatmap results showed that Y1 and Y2, G2 and G3, and G1 and CK have similar expression patterns. The numbers of genes exhibiting either upregulation or downregulation between two samples were depicted in Figure 3C, and the numbers of DEGs between Y1 and Y2, G2 and G3, and G1 and CK were relatively small. Taken together, these results suggested that the responses of small leaves at different positions to the PMV displayed a marked difference and PMV infection was less severe in newer green leaves (G1 sample). To validate the RNA sequencing data, ten key DEGs for this paper were selected for qRT-PCR analysis. The qRT-PCR results indicated that all of these DEGs exhibited similar expression kinetics to those obtained from the RNA sequencing analysis (Figure S4), thus supporting the validity of the method used for determining DEGs from the RNA sequencing analysis.

Gene ontology (GO) functional classification included 3 GO trees (cellular components, molecular functions, and biological processes) and 49 functional groups (Table S9). In the category of molecular function, the largest groups were catalytic activity, binding and transporter activity. For the cellular components, DEGs with cell, cell part, membrane, membrane part, and organelle formed the major groups. Metabolic process, cellular process, single-organism process, regulation of biological process, response to stimulus, localization, biological regulation, and cellular component organization or biogenesis were the dominant groups in the category of biological processes (Table S9).

**Figure 4:**
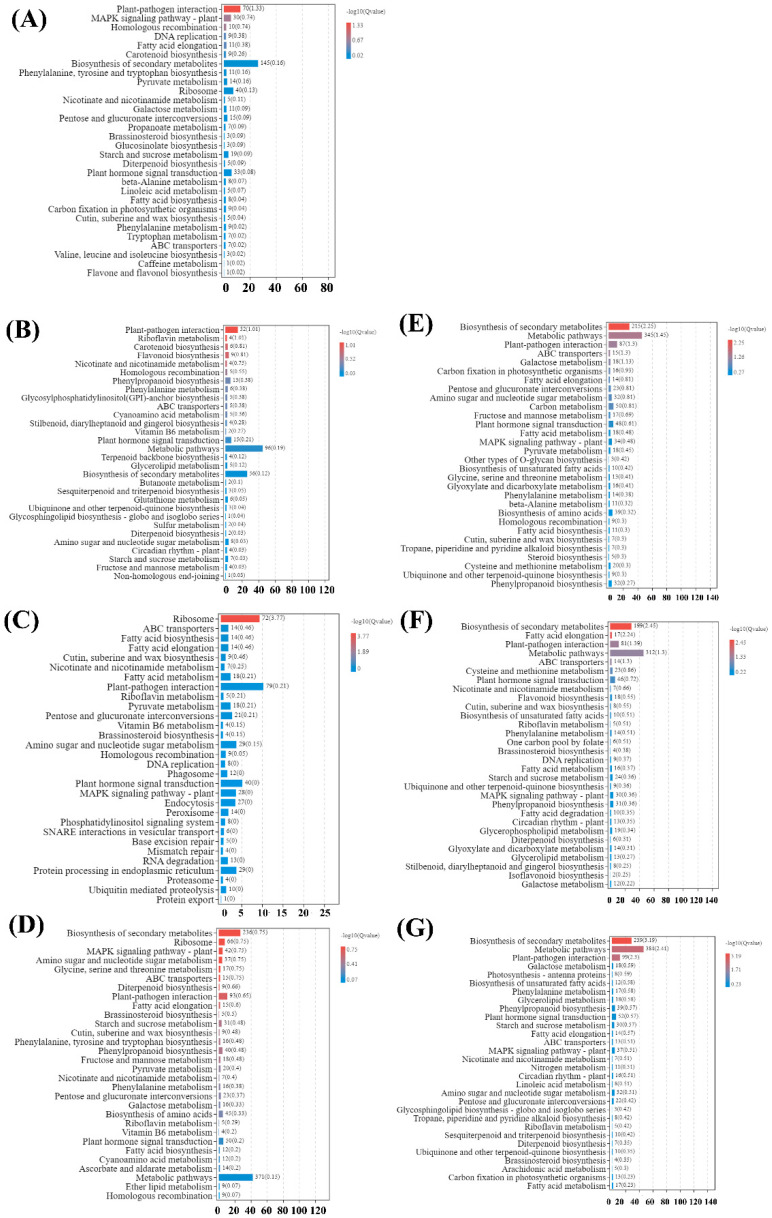
Top 30 KEGG pathways enrichment analysis of DEGs in PMV-infected plants leaves versus CK. (**A**) CK vs. ML, (**B**) CK vs. G1, (**C**) CK vs. G2, (**D**) CK vs. G3, (**E**) CK vs. Y1, (**F**) CK vs. Y2, (**G**) CK vs. Y3.

In order to understand their biological function, all of the DEGs were also mapped to terms in the Kyoto Encyclopedia of Genes and Genomes (KEGG) database. The results showed that the significantly enriched pathways were different among CK vs. ML, CK vs. G1, CK vs. G2, CK vs. G3, CK vs. Y1, CK vs. Y2, and CK vs. Y3 (Figure 4 and Table S10). Moreover, the KEGG pathways enrichment analysis of DEGs was also performed among G1 vs. G2, G2 vs. G3, G1 vs. G3, Y1 vs. Y2, Y1 vs. Y3, Y2 vs. Y3, G1 vs. Y1, G2 vs. Y2, and G3 vs. Y3 in PMV-infected plant leaves (Table S11). The results showed that there were differences in KEGG enrichment of DEGs among different comparison groups. The pathways, including plant–pathogen interaction, MAPK signaling pathway—plant, ribosome, fatty acid biosynthesis, fatty acid elongation, starch and sucrose metabolism, plant hormone signal transduction, photosynthesis, and linoleic acid metabolism were dominant pathways and should be focused on elucidating the molecular mechanism of pecan response to PMV.

### 2.5. Inferred Pecan-PMV Interaction Pathway Suggests PTI and ETI

**Figure 5:**
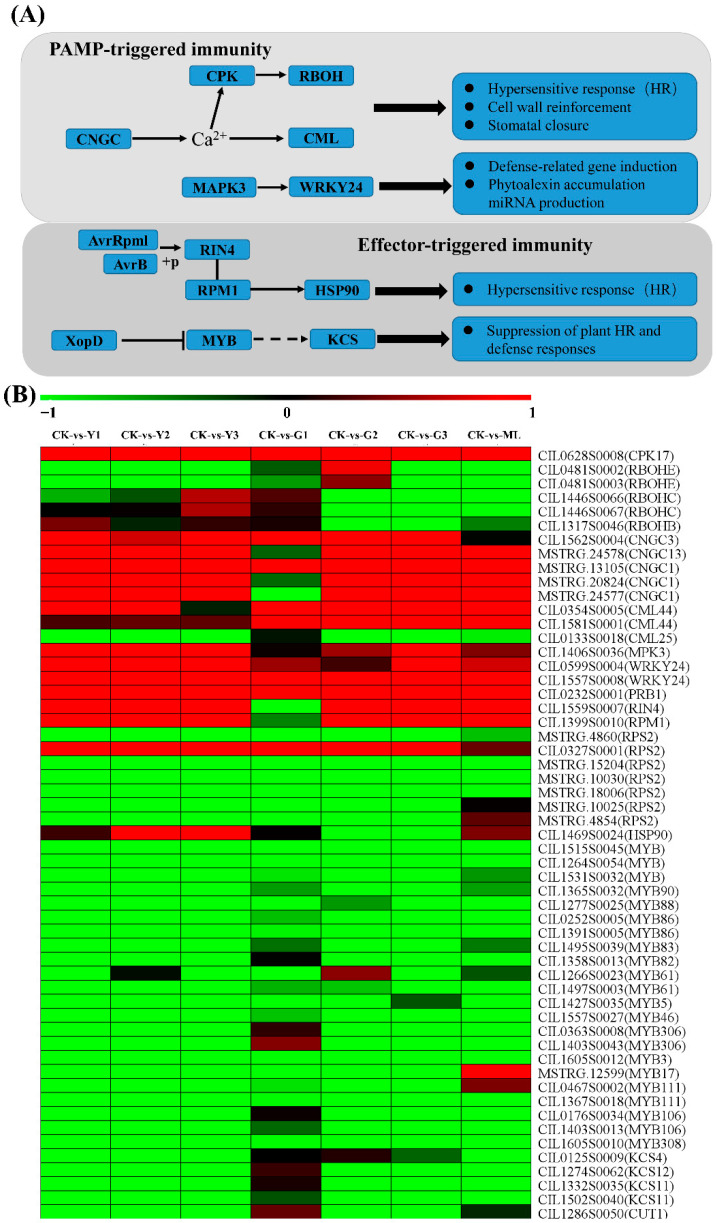
Inferred pecan-PMV interaction pathway using KEGG ortholog terms (**A**) and heat map of DEGs (**B**). Responsive progression of gene expression under different samples infected PMV compared to CK. Heat maps were drawn using the Log2 ratio (transformed FPKM value of PMV infection/those of CK). Detailed genes and expressions data were provided in Table S12, available as Supplementary Data online. CNGC, cyclic nucleotide-gated channel; CPK, calcium-dependent protein kinase; CML, calcium-binding protein; MPK3, mitogen-activated protein kinase 3; WRKY24, WRKY transcription factor 24; RBOH, respiratory burst oxidase homolog protein; PR1, pathogenesis-related protein 1; RIN4, RPM1-interacting protein 4; RPM1, disease resistance protein RPM1-like; RPS, probable disease resistance protein; HSP90, heat shock protein 90; EDS1: enhanced disease susceptibility 1 protein; MYB, transcription factor MYB; KCS or CUT, 3-ketoacyl-CoA synthase.

DEGs were mapped to the plant–pathogen interaction reference pathway (ko04626) deposited in the KEGG database (Figure 4) to infer the signaling pathway of pecan in response to PMV attack. A total of 153 DEGs were identified from infected plants compared to the CK (Figure 5 and Table S12). According to the pathway function, plenty of genes encoding calcium-dependent protein kinase (CPK), cyclic nucleotide-gated channel (CNGC), calcium-binding protein (CML), mitogen-activated protein kinase 3 (MPK3), WRKY transcription factor, pathogenesis-related protein 1 (PR1) were upregulated in PMV-infected plant compared to CK, suggesting that most identified genes were involved in the perception of the pathogen by PTI. RPM1-interacting protein 4 (RIN4) and disease resistance protein RPM1-like (RPM1) genes expression were upregulated in PMV-infected plant compared to CK, indicating ETI was involved in pecan in response to PMV infection. However, most genes coding MYB transcription factor and 3-ketoacyl-CoA synthase (KCS) were downregulated in PMV-infected plants (Figure 5 and Table S12).

### 2.6. Identified Differentially Expressed Plant Disease Resistance (R) Protein Genes in Pecan in Response to PMV

**Figure 6:**
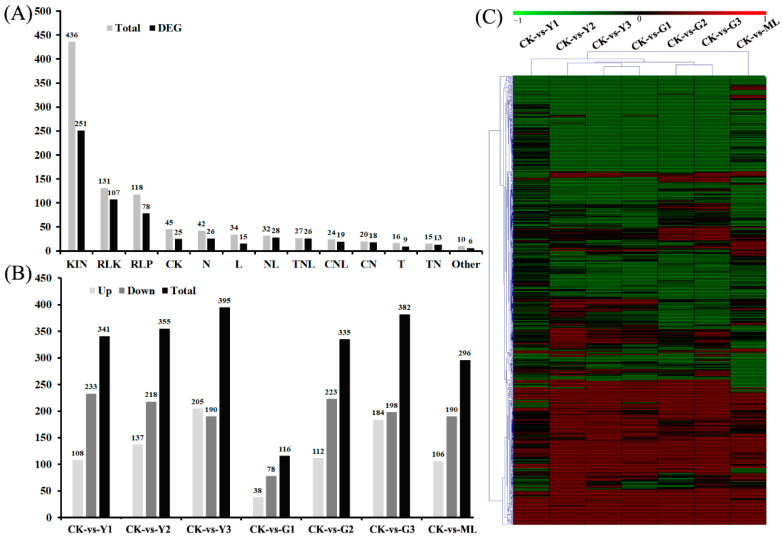
Differentially expression of R genes in pecan response to PMV. (**A**) Classification and quantity of predicted pecan R proteins. (**B**) Differentially expressed R genes in different infection leaves compared to CK. (**C**) Heatmap of differential expression of R genes. Responsive progression of gene expression under different samples infection PMV compared to CK. Heat maps were drawn using the Log2 ratio (transformed FPKM value of PMV infection/those of CK). Detailed gene and expression data are provided in Table S13, available as Supplementary Data online.

All of the protein domains of the detected genes were predicted by Pfam, and six typical disease resistance-related protein domains, including the coiled-coil (CC) domain, Tollinterleukin 1 receptor (TIR) domain, leucine-rich repeat (LRR) domain, nucleotide binding site (NBS) domain, and transmembrane (TM) domain and kinase domain were analyzed according to the previous reports [22,23,24]. These domains play a significant role in R protein interactions with effector proteins from pathogens and in activating signal transduction pathways involved in innate immunity. In total, 950 R genes and 621 differentially expressed R genes were identified in pecan in response to PMV, including KIN (kinase), RLK (LRR, TM, and kinase), RLP (TM and LRR), CK (CC, TM, and kinase), N (NBS), L (LRR) NL (NBS, TM, and kinase), TNL (TIR, NBS, and LRR), CNL (CC, NBS, and LRR), CC (CC, NBS, and TM), T (TIR), TN (NBS, TM, and TIR), and other (Figure 6 and Table S13). However, the expression profiles among seven samples compared to the CK showed that these genes were up- or downregulated (Figure 6 and Table S13), suggesting their different responses to the PMV infection.

### 2.7. PMV Infection Downregulated the Genes Related to the Photosynthesis

**Figure 7:**
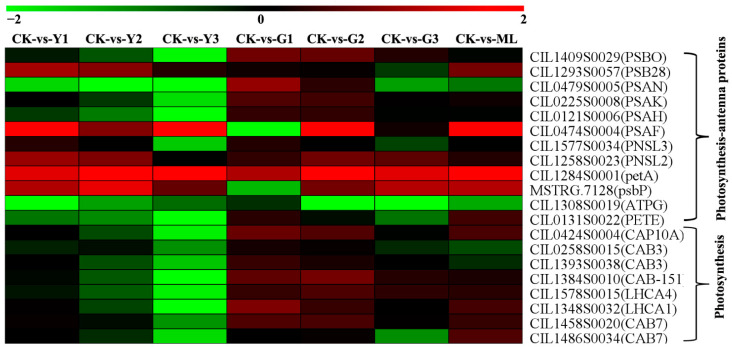
Heatmap of DEGs of photosynthesis and photosynthesis-antenna pathways. Responsive progression of genes expressions under different samples infection PMV compared to CK. Heat maps were drawn using the Log2 ratio (transformed FPKM value of PMV infection/those of CK). Detailed gene and expression data are provided in Table S14, available as Supplementary Data online.

A total of 20 DEGs associated with the photosynthesis pathway were identified in PMV-infected plants compared to CK (Figure 7 and Table S14), including Photosystem I (4), Photosystem II (4), cytochrome b6-f complex (1), photosynthetic electron transport (1), F-type ATPase (1), and photosynthesis-antenna proteins (8). The expressions of *PsaH*, *PsaK*, and *PsaN* in PSI, *PsbO*, *PSBQ* in PSII, *petE* in the photosynthetic electron transport, and *atpB* in F-type ATPase-related were downregulated significantly in PMV-infected pecan yellow leaves (Y3), and their expression decreased gradually with Y1, Y2, and Y3 except for the *atpB.* In addition, all of the detected chlorophyll a-b binding proteins related to photosynthesis-antenna proteins-related genes were decreased gradually with Y1, Y2, and Y3 and downregulated significantly in Y3 (Figure 7). However, their expressions were no obvious change in green leaves and mottle leaves.

### 2.8. DEGs Analysis of Plant Hormone Biosynthesis and Signal Transduction Pathways

**Figure 8:**
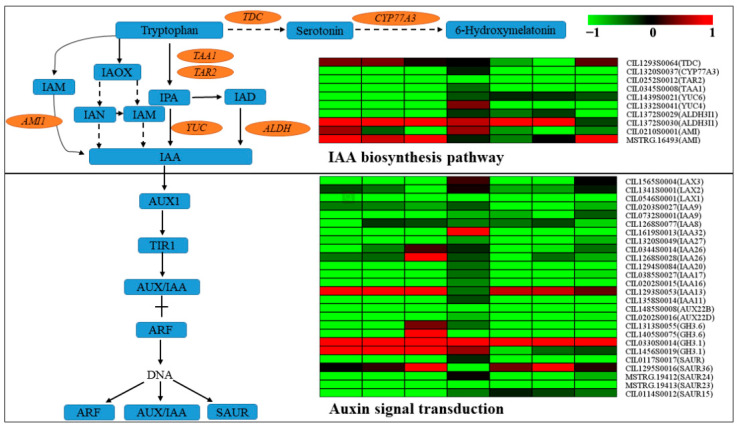
DEGs involved in AUX biosynthesis pathways and signaling transduction pathways. Responsive progression of genes expressions under different samples infection with PMV compared to the control (CK), indicated in 7-box strings (including Y1-vs.-CK, Y2-vs.-CK, Y3-vs.-CK, G1-vs.-CK, G2-vs.-CK, G3-vs.-CK, ML-vs.-CK). Heat maps were drawn using the Log2 ratio (transformed FPKM value of PMV infection/those of CK). Detailed genes and expressions data were provided in Tables S15 and S16, available as Supplementary Data online. TAA1, tryptophan aminotransferase of Arabidopsis 1; TAR2, tryptophan aminotransferase related 2; IAOx, indole-3-acetaldoxime; IAM, indole-3-acetamide; IAN, indole-3-acetonitrile; AMI, indole-3-acetamide hydrolase; IPA, indole-3-pyruvic acid; IAA, indole-3-acetic acid; TDC, tryptophan decarboxylase; IAD, indole-3-acetaldehyde; ALDH, aldehyde dehydrogenase; YUC, indole-3-pyruvate monooxygenase YUCCA; ARF, auxin response factors; LAX, auxin transporter-like protein; IAA, auxin-responsive protein IAA; GH3, indole-3-acetic acid-amido synthetase GH3; SAUR, auxin-responsive protein SAUR; TIR, transport inhibitor response. The heat maps indicated the upregulation (red) and downregulation (green) of genes in PMV-infected samples compared to CK.

**Figure 9:**
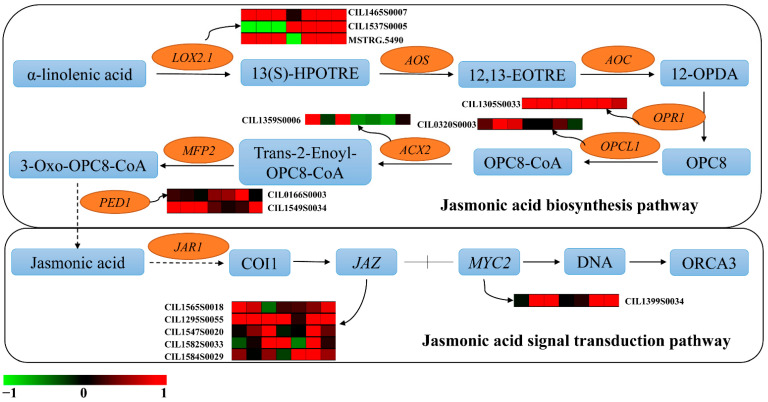
DEGs involved in jasmonic acid (JA) biosynthesis and signal transduction pathways. Responsive progression of genes expressions under different samples infection with PMV compared to CK, indicated in 7-box strings (including Y1-vs.-CK, Y2-vs.-CK, Y3-vs.-CK, G1-vs.-CK, G2-vs.-CK, G3-vs.-CK, ML-vs.-CK). Heat maps were drawn using log2 (transformed FPKM value of PMV infection/those of CK). Detailed gene and expression data are provided in Tables S15 and S17, available as Supplementary Data online. LOX, lipoxygenase; AOS, allene oxide synthase; AOC, allene oxide cyclase; ACX, acyl-CoA oxidase; PED1, 3-ketoacyl-CoA thiolase 2, peroxisomal-like; JAR1, jasmonate resistant 1; OPR, 12-oxophytodienoic acid reductase gene; OPCL1, OPC-8,0 CoA ligase gene; 13(S)-HPOTRE, (9Z,11E,15Z)-(13S)-13-Hydroperoxyoctadeca-9, 11,15-trienoic acid; 12,13(S)-EOTRE, (9Z,15Z)-(13S)-12,13-Epoxyoctadeca-9,11,15-trienoic acid; 12-OPDA, (15Z)-12-Oxophyto-10,15-dienoic acid; OPC8, 8-[(1R,2R)-3-Oxo-2-(Z)-pent-2-enyl cyclopentyl]octanoate; MYC2, transcription factor MYC2; JAZ, jasmonate ZIM domain-containing protein. The heat maps indicated the upregulation (red) and downregulation (green) of genes in PMV-infected samples compared to CK.

**Figure 10:**
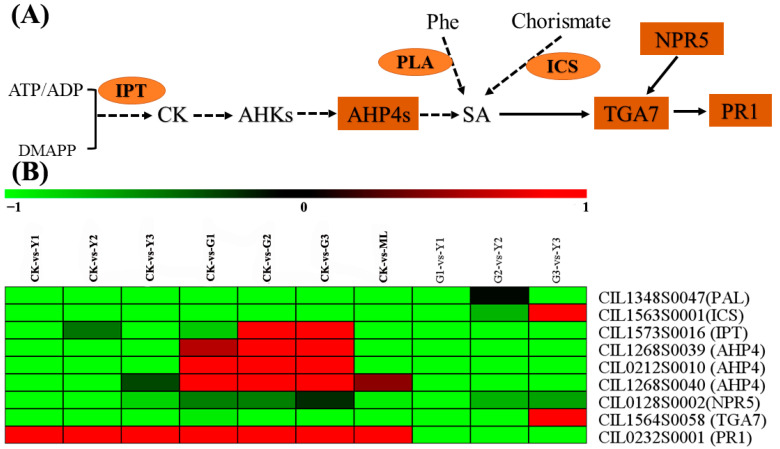
Model of cytokinin and SA action in response to PMV infection (**A**) and DEGs (**B**). Heat maps were drawn using the Log2 ratio. Detailed genes and expressions data are provided in Table S15, available as Supplementary Data online. PAL, phenylalanine ammonia-lyase; Phe, phenylalanine; ICS, isochorismate synthase; ATP/ADP, adenosine 5′-phosphates; DMAPP, dimethylallyl diphosphate; AHK, histidine kinase; AHP, histidine-containing phosphotransfer protein; SA, salicylic acid; TGA7, transcription factor TGA7; NPR5, non-expressor of PR gene 5; PR1, pathogenesis-related protein 1; IPT, adenylate isopentenyltransferase. The heat maps indicated the upregulation (red) and downregulation (green) of genes in PMV-infected samples compared to CK.

Plant hormone signal transduction pathway (Ko04075) was enrichment in PMV-infected pecan plants using KEGG analysis (Table S15). So, the DEGs of plant hormone biosynthesis and signal transduction pathways were analyzed. As for the AUX biosynthesis and signal transduction pathways (Figure 8 and Table S16), *Tryptophan aminotransferase of Arabidopsis 1* (*TAA1*), *Tryptophan aminotransferase related 2* (*TAR2*), *Indole*-*3*-*pyruvate monooxygenase YUCCA* (*YUCs*), and *Aldehyde dehydrogenase* (*ALDH*) transcripts were decreased significantly in PMV-infected plants, and numerous of the auxin-signaling *IAA* genes were also downregulated except for CIL1293S0053. However, the expressions of *GH3.1s* (CIL0330S0014 and CIL1456S0019) were upregulated in PMV-infected plants, especially in the yellow leaves. As for the JA biosynthesis and signal transduction pathways (Figure 9 and Table S17), the expressions of JA biosynthesis genes, including *lipoxygenase 2.1s* (*LOX 2.1s*), *acyl*-*CoA oxidase 2*(*ACX2*), *3*-*ketoacyl*-*CoA thiolase 1*(*PED1*), *12*-*oxophytodienoic acid reductase gene*(*OPR*), and *OPC*-*8,0 CoA ligase gene* (*OPCL1*) and JA signal transduction genes, including *transcription factor MYC2* (*MYC2*) and *jasmonate ZIM domain*-*containing protein* (*JAZ*) were upregulated obviously in PMV-infected pecan plants. The *adenylate isopentenyltransferase* gene (*IPT*, CIL1573S0016), which was the key gene of cytokinin biosynthesis, was downregulated significantly in yellow leaves but upregulated significantly in green leaves (G2 and G3 samples) of PMV-infected pecan plants. Correspondingly, three *histidine*-*containing phosphotransfer protein 4* genes (*AHP4*) had the same expression patterns (Figure 10 and Table S15). These results showed that cytokinin biosynthesis and signaling transductions were enhanced in green leaves but restrained in yellow leaves of PMV-infected pecan plants. For SA biosynthesis, the expression of *isochorismate synthase* (*ICS*, CIL1563S0001) and *phenylalanine ammonia*-*lyase* (*PAL*, CIL1348S0047) genes were both downregulated significantly in all samples in PMV-infected pecan plants, while the expression levels of PAL in green leaves higher than those in yellow leaves. Similarity, the *non*-*expressor of PR5* gene (*NPR5*, CIL0128S0002) and *transcription factor TGA7* (TGA7, CIL1564S0058) genes had same expression patterns with *PAL*. Moreover, the *pathogenesis*-*related protein 1* (*PR1*, CIL0232S0001) gene transcript was increased remarkable in all samples, and the expression level of *PR1* in green leaves was higher than that in yellow leaves (Figure 10 and Table S15). A few genes referred to the gibberellin, abscisic acid, ethylene, and brassinosteroids signal transduction pathways were changed obviously in PMV-infected pecan plants (Table S15).

### 2.9. DEGs of Fatty Acid Biosynthesis, Elongation, and Degradation Pathways

**Figure 11:**
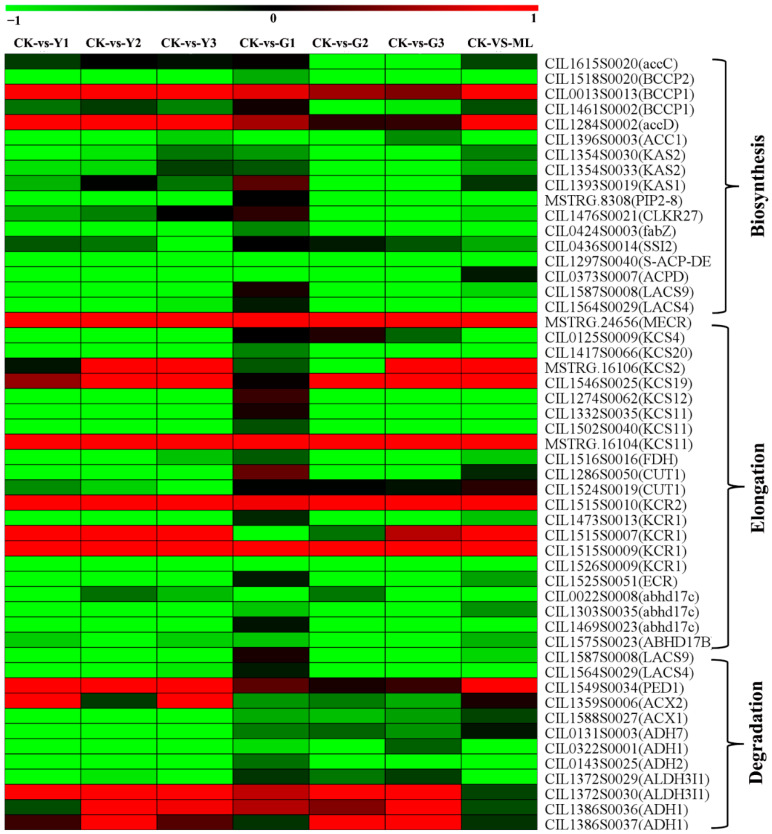
DEGs involved in fatty acid biosynthesis, elongation, and degradation pathways. Responsive progression of genes expressions under different samples infection PMV compared to CK. Heat maps were drawn using the Log2 ratio (transformed FPKM value of PMV infection/those of CK). Detailed gene and expression data are provided in Table S18, available as Supplementary Data online. The heat maps indicated the upregulation (red) and downregulation (green) of genes in PMV-infected samples compared to CK.

Fatty acid biosynthesis (Ko00061), elongation (Ko00062), and metabolism (Ko01212) pathways were detected through KEGG analysis (Figure 4). A total of 17 DEGs of fatty acid biosynthesis were altered significantly in PMV-infected samples compared to CK, and 15 genes were downregulated, including genes coded acetyl-CoA carboxylase 1 (ACC1), 3-oxoacyl-acyl-carrier-protein synthase (KAS), stearoyl-acyl-carrier-protein 9-desaturase (SSI2, ACPD), and so on. A total of 24 DEGs of fatty acid elongation were changed significantly, and 17 genes were downregulated, including eight genes coded 3-ketoacyl-CoA synthase (KCSs, FDH, and CUTs), two genes coded very-long-chain 3-oxoacyl-CoA reductase (KCR) and a very-long-chain enoyl-CoA reductase (ECR) (Figure 11 and Table S18). Moreover, many MYB transcription factors were detected, and most of these genes were downregulated (Figure 11 and Table S12). These results showed that the fatty acid and very-long-chain fatty acids (VLCFAs) biosynthesis were blocked in PMV-infected pecan plants.

### 2.10. DEGs Involved in Starch and Sucrose Metabolism Pathway

**Figure 12:**
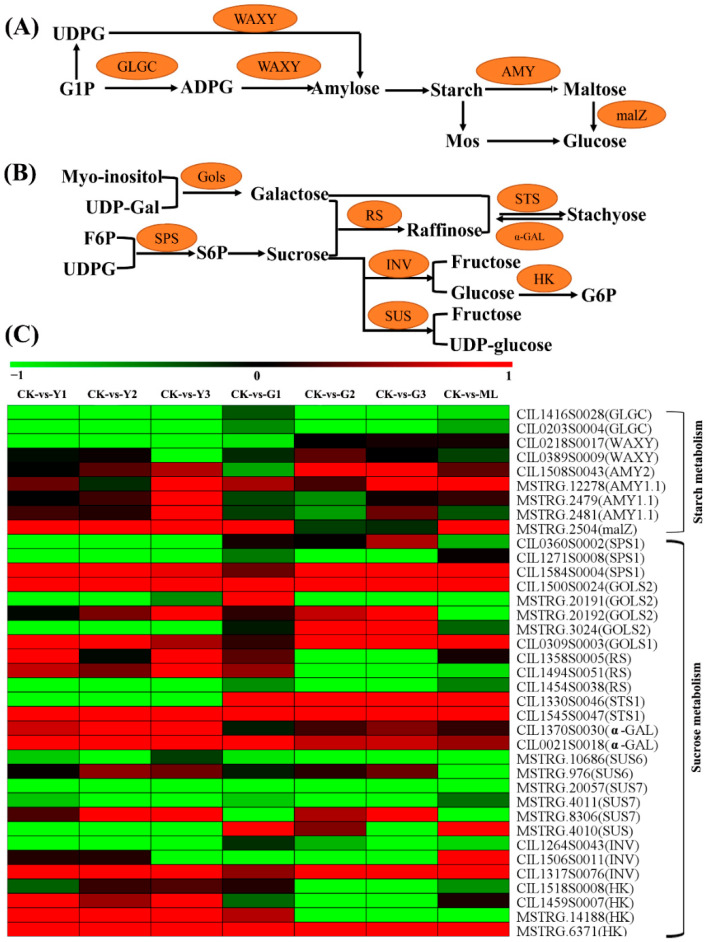
Carbohydrate metabolic pathways. (**A**) Starch biosynthesis and degradation pathway. (**B**) Sucrose biosynthesis and metabolism pathway. (**C**) Heatmap of DEGs of carbohydrate metabolic pathway. Responsive progression of genes expressions under different samples infection with PMV compared CK. Heat maps were drawn using the Log2 ratio (transformed FPKM value of PMV infection/those of CK). Detailed genes and expressions data were provided in Table S19, available as Supplementary Data online. WAXY, granule-bound starch synthase; GLGC, glucose-1-phosphate adenylyltransferase; AMY, alpha-amylase; malZ, alpha-glucosidase; UDPG, UDP-glucose; ADPG, ADP-glucose; G1P, glucose-1P; F6P, fructose 6-phosphate; RS, raffinose synthase; SPS, sucrose phosphate synthase; SUS, sucrose synthase; S6P, sucrose-6-phosphate; HK, hexokinase; STS, stachyose synthase; UDPG, UDP-glucose; UDP-Gal, UDP-galactose; GOLS, galactinol synthase. α-GAL, alpha-galactosidase; INV, beta-fructofuranosidase. The heat maps indicated the upregulation (red) and downregulation (green) of genes in PMV-infected samples compared to CK.

To predict important candidate genes for carbohydrate metabolism under PMV attack conditions, we screened PMV-responsive genes for those encoding enzymes involved with starch biosynthesis and degradation, sucrose metabolism. The abundance of several of the transcripts encoding starch biosynthesis and degradation enzymes was elevated in pecan plants after exposure to PMV attack (Figure 12 and Table S19). The genes encoding glucose-1-phosphate adenylyltransferase (GLGC) were downregulated in PMV-infected pecan plants. The transcripts level of genes encoding the granule-bound starch synthase (WAXYs) was downregulated in yellow levels of PMV-infected pecan plants, especially in the Y3 sample. In particular, the levels of three alpha-amylase transcripts were significantly upregulated in the Y3 sample. The transcripts of sucrose metabolism genes encoding galactinol synthase (GOLS), sucrose phosphate synthase (SPS), raffinose synthase (RS), beta-fructofuranosidase (INV), sucrose synthase (SUS), stachyose synthase (STS), alpha-galactosidase (α-GAL) and hexokinase (HK) were changed obviously in PMV-infected plants compared to CK. Among genes encoding alkaline/neutral invertases, PMV infection induced the cell wall INV gene (CIL1317S0076) expression, suppresses the acid INV gene (CIL1264S0043) expression, and alters the insoluble CWINV1 (CIL1506S0011) expression. Moreover, the expression levels of the cell wall INV gene (CIL1317S0076) in yellow leaves were higher than those in green leaves in PMV-infected pecan plants, especially in the Y3 sample (Figure 12 and Table S19).

### 2.11. DEGs of Ribosome and Ribosome Biogenesis in Eukaryotes Pathways

**Figure 13:**
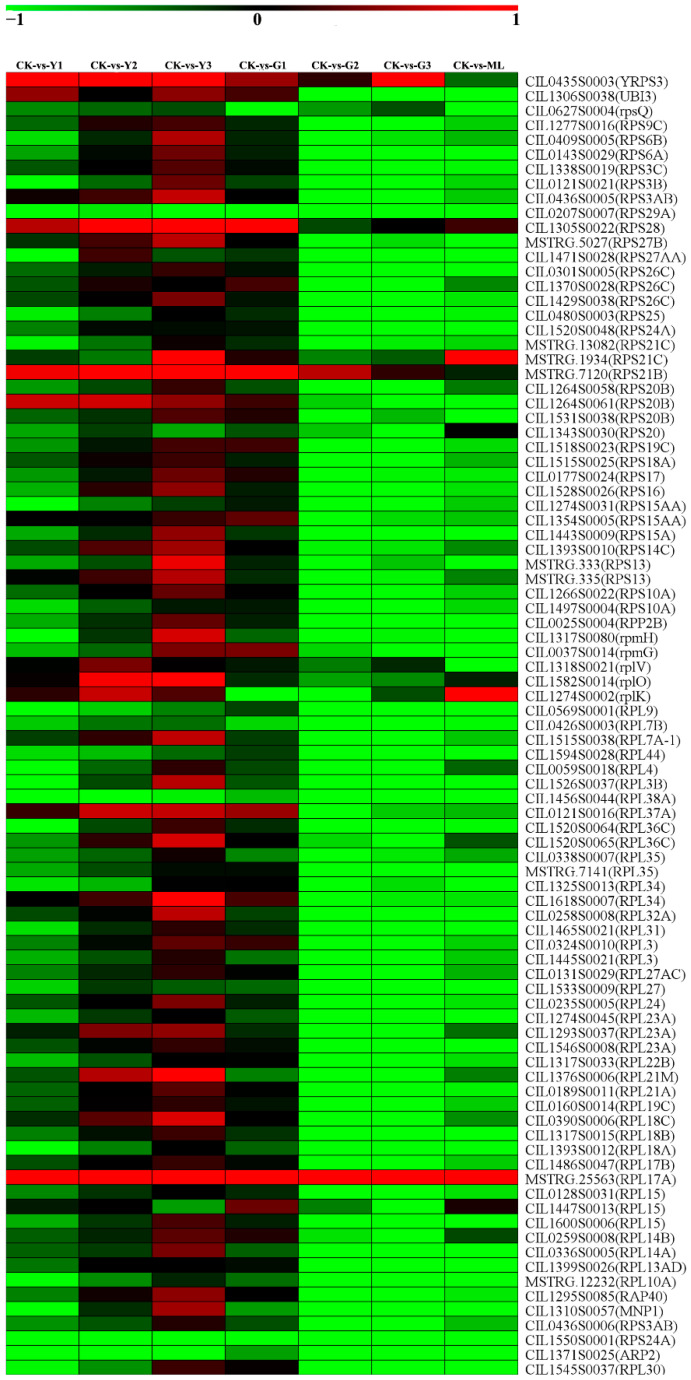
Heat map of DEGs of ribosome pathways (Ko03010). Responsive progression of genes expressions under different samples infection PMV compared to CK. Heat maps were drawn using the Log2 ratio (transformed FPKM value of PMV infection/those of CK). Detailed genes and expressions data are provided in Table S20, available as Supplementary Data online. The heat maps indicated the upregulation (red) and downregulation (green) of genes in PMV-infected samples compared to CK.

The ribosome pathway was significantly enriched in G2 and G3 samples compared to CK (Figure 13 and Table S20)**.** The DEGs of ribosome and ribosome biogenesis in eukaryotes pathways were analyzed in our study. A total of 89 DEGs related to the ribosome pathway (Ko03010) were obtained in PMV-infected samples compared to CK (Figure 13 and Table S20). A total of 80 out of 89 40S, 60S, and other ribosomal protein genes were downregulated significantly in G2 and G3 samples. A total of 13 ribosomal protein genes were downregulated significantly in the Y1 sample, and the expressions of these genes were increased significantly gradually with Y1, Y2, and Y3. The expressions of almost all genes were not changed significantly in Y2, Y3, and G1 samples. The expressions of 79 ribosomal protein genes in the Y2 and Y3 samples were significantly higher than those in G2 and G3 samples. These results showed that the expressions of ribosomal proteins were significantly downregulated in green leaves, whereas there was no obvious change in yellow leaves of PMV-infected pecan plants.

A total of 19 DEGs of ribosome biogenesis in eukaryotes (Ko03008) were obtained in PMV-infected samples compared to CK (Table S21). A total of 11 out of 19 genes were downregulated significantly in G2 and G3 samples. Eight gene expressions were downregulated significantly in the Y1 sample. The expressions of all 19 genes were increased gradually with Y1, Y2, and Y3. The expressions of 18 ribosome biogenesis genes in the Y2 and Y3 samples were higher than those in G2 and G3 samples. Similar to ribosome protein gene expression patterns, expressions of ribosome biogenesis genes were significantly downregulated in green leaves, whereas there were no obvious changes in yellow leaves of PMV-infected pecan plants. Taken together, we deduced that disturbance of the ribosome biogenesis might enhance the resistance to PMV infection in pecan and lead to leaves staying green.

## 3. Discussion

### 3.1. PMV-Infected Pecan Trigged PTI and ETI

Plants defense against invading pathogens rely two layers innate immune system, PTI and ETI [25,26]. PTI was activated by plant pattern recognition receptors (PRRs), and ETI was activated by plant R protein. In most cases, PTI efficiently prevented pathogens from infecting the plant without any visible symptoms [27]. The major feature of ETI was its robustness against pathogen infection, which was frequently associated with hypersensitive response (HR) cell death [2,4].

In our study, there were 153 DEGs associated with the plant–pathogen interaction pathway in PMV-infected pecan leaves compared to CK leaves (Figure 5 and Table S12). Plenty of R proteins were up- or downregulated in pecan response to PMV (Figure 6 and Table S13). By mapping the KO terms attached to the identified DEGs to the KEGG plant–pathogen interaction pathway, the innate immune pathway, including PTI and ETI, was deducted, suggesting that a series of downstream signaling responses, including a Ca^2+^ burst and the MAPK cascade, were triggered in pecan response to PMV attack and lead to cell wall reinforcement, stomatal closure, and HR. The specific disease resistance proteins (RIN4, RPM1) could contribute to the detection of the pathogen-secreted effector proteins, followed by activation of HR.

RPM1 was a CC-NB-LRR class R protein that was shown to be required for resistance to pathogens infection in *Arabidopsis* [28] and wheat [29]. RIN4 interacts with RPM1, AvrB, and AvrRpm1, and was required for the RPM1 accumulation and function. However, RIN4 could negatively regulate basal defense responses, since the *rin4* mutant, which constitutively expresses PR1 and PR5, displayed resistance against virulent pathogens. AvrRpm1 and AvrB phosphorylated RIN4 in the membrane fraction of the plant cell. The fraction of AvrRpm1 and AvrB sufficient to induce phosphorylation of RIN4 might constitute the same fraction associated with RIN4. Manipulation of RIN4 might enhance the basal defenses of the plant. AvrB induced RIN4 protein levels by increasing transcription of *RIN4*. RPM1 might “guard” against pathogens that used AvrRpm1 and AvrB to manipulate RIN4 activity [28]. In our study, the expressions of RIN4 and RPM1 were induced by PMV in pecan, showed that the same as *Arabidopsis*, RIN4 was required for RPM1-mediated resistance in pecan, and induced HR.

### 3.2. ETI or PTI Might Induce Activation of MAPK and Inhibited Photosynthesis, Leading to Leaf Chlorosis

Despite the different recognition and activation mechanisms of NLRS and PRRs, ETI and PTI involved a similar set of downstream defense responses, including the Ca^2+^-mediated signaling, activation of MAPKs, and production of ROS. ETI was proposed to be an amplified PTI [4]. Activation of tobacco SA-induced protein kinase (SIPK) and wound-induced protein kinase (WIPK), respectively, inhibited photosynthesis and induced the accumulation of ROS in chloroplasts, which accelerated HR-like cell death in plants under light [30]. Pathogen infection inhibited photosynthesis, including inhibition of Photosystem II activity, reduction in CO_2_ fixation, and global downregulation of photosynthetic genes.

*Arabidopsis* MPK3 and MPK6, which were rapidly activated during PTI and ETI, played critical roles in multiple plant defense responses, including activation of defense gene expression, induction of phytoalexin biosynthesis, and stomatal immunity [31,32]. Downregulation of photosynthetic genes commonly occurred in plants under biotic attack according to previous comparative transcriptome analyses [33], which might be because ETI induced prolonged activation of MAPK, thus inhibiting photosynthesis [34]. *Arabidopsis* MPK3/MPK6 activation induced a global downregulation of genes related to photosynthesis in chloroplasts (including photosynthetic light harvesting, light reaction, electron transport, and dark reaction) along with an upregulation of defense-related genes, and coordinated the growth and defense trade-off in plants [34], indicating that MPK3/MPK6-mediated active photosynthetic inhibition was a part of *Arabidopsis* immune response and played a positive role during ETI. Active photosynthetic inhibition mediated by MPK3/MPK6 was critical to effector-triggered immunity in *Arabidopsis* [34].

It was interesting that the expressions of *MPK3* (CIL1406S0036) were upregulated significantly in yellow leaves (Y1, Y2, Y3), and their expression increased gradually with Y1, Y2, and Y3. Whereas their expressions were no obvious change in green leaves (G1, G2, and G3) and ML leaves (Table S12). Correspondingly, the photosynthesis and photosynthesis-antenna proteins-related gene expressions were downregulated in PMV-infected plants’ yellow leaves and decreased gradually with Y1, Y2, and Y3 (Table S14). These results showed that the activation of MPK3 by PMV inhibited the expressions of photosynthesis-related genes and caused leaf chlorosis in pecan. Inhibition of photosynthesis after the activation of pathogen-responsive MPK3/MPK6 in *Arabidopsis* or their orthologs in other plant species was a common response in plants [34]. We concluded that MPK3-mediated active photosynthetic inhibition was a part of the pecan immune response and played a positive role during ETI. Meanwhile, the expressions of *MAPKKK17* (CIL0135S0010) and *MKK9* (CIL0149S0012) were upregulated significantly in yellow leaves (Y1, Y2, and Y3), and their expressions were significantly higher than those in green leaves (Table S8). Thus, the MAPKKK17-MKK9-MPK3 cascade reaction might be involved in the process of resistance to PMV infection and need further experimental verification.

### 3.3. PMV Impairs Chloroplasts Clustering to Facilitate Viral Infection

Chloroplasts were one of the most dynamic organelles in land plants. It not only fixed carbon through photosynthesis but also synthesized key biochemical components, including amino acids, fatty acids, purine, and pyrimidine. Moreover, chloroplasts played vital roles in plant defense against microbial plant pathogens [6,35]. Plant viruses could replicate and assemble their virions efficiently utilizing host resources, and viral proteins interact with and exploit chloroplast proteins in order to support viral cell-to-cell movement. Upon viral infection, chloroplasts undergo enormous structural and functional damage [36]. The chloroplasts were a prime target for viruses, and damage to the chloroplasts was one of the pivotal steps for viruses’ successful infection. Viruses could exploit the chloroplast’s double-membrane structure for propagation. Turnip yellow mosaic virus (TYMV) of the *Tymoviridae* family and Turnip mosaic virus (TuMV) of the *Potyviridae* family were regularly associated with the chloroplast’s membrane during infection [7,37]. The existing evidence suggested that some plant RNA viruses required an appropriate lipid composition, together with sufficient fluidity and plasticity of the membrane for replication [38].

Indeed, large proportions of affected gene products in a virus-infected plant were closely associated with the chloroplast and the process of photosynthesis [6]. Sugarcane mosaic virus (SCMV) infection significantly downregulated chloroplast *violaxanthin deepoxidase* (*VDE*) mRNA levels in the early stage in maize. ZmVDE interacted with SCMV HC-Pro and attenuated the RSS activity of SCMV HC-Pro, played a positive role against SCMV infection [39]. Chloroplast NADH dehydrogenase-like (NDH) complex M subunit gene (*NdhM*) was first upregulated and then downregulated in turnip mosaic virus (TuMV)-infected *Nicotiana benthamiana*. Upon perception of TuMV invasion, *NbNdhM* was rapidly induced to trigger chloroplasts clustering around nuclei. Overexpression of *NbNdhM* significantly induced the clustering of chloroplasts around the nuclei, and silencing of *NbNdhM* facilitated TuMV infection, suggesting that the chloroplasts clustering mediated by *NbNdhM* was a defense against TuMV [40]. The chloroplast protein ferredoxin 1 (FD1) interacted with the coat proteins (CPs) of tomato mosaic virus (TMV), cucumber mosaic virus (CMV) and potato virus X (PVX). *FD1* mRNA and protein levels were reduced by PVX infection. Overexpression of *FD1* manifested resistance to PVX infection, silencing of FD1 significantly increases PVX accumulation [41]. Tobacco mosaic virus infection of *N*. *tabacum* plants resulted in 50% reductions in ATP-synthase g-subunit (AtpC) and rubisco activase (RCA) messenger RNA levels. In AtpC- and Rca-silenced leaves, TMV accumulation and pathogenicity were greatly enhanced, suggesting a role of both host-encoded proteins in a defense response against TMV. The host chloroplast proteins AtpC and RCA interacted with TMV replication-associated proteins and VRCs in vitro and played a role in plant defense specifically against tobacco mosaic virus [42]. The expression of *ferredoxin oxidoreductase* (HY, CIL1359S0027) and *violaxanthin deepoxidase* (CIL1312S0049) genes were upregulated in yellow leaves, rather than not changed in green leaves by PMV infection. On the other hand, the expressions of photosynthesis and photosynthesis-antenna proteins-related genes were downregulated in yellow leaves of PMV-infected plants, and decreased gradually with Y1, Y2, and Y3. Meanwhile, the cell and chloroplasts structures were severely damaged in yellow leaves of PMV-infected pecan plants. Plant healthy photosynthetic tissue was often a requirement for virus infection. Viruses make use of the energy stored inside carbon compounds biosynthesized by chloroplasts for their survival and propagation. Viruses regulate sugar efflux, carbon partition, and phloem transport of metabolites, increasing the need for photosynthesis inside the host cell. Nevertheless, the enhanced activity and vigor of the organelle also increased the threat of the antiviral immunity response [6]. The half leaves of PMV-infected pecan plants stayed green (Figure 1) and triggered chloroplasts clustering (Figure 2), suggesting that the green leaves of PMV-infected pecan plants could establish a strong antiviral immunity system.

### 3.4. The Involvement of the JA Pathway in Auxin-Mediated Defense against PMV Infection

Much research has shown that auxin plays an important role in plant defense against various pathogen infections. Trp-dependent IAA biosynthesis had four pathways, including the indole-3-acetamide (IAM) pathway, the indole-3-pyruvic acid (IPA) pathway, the tryptamine (TAM) pathway, and the indole-3-acetaldoxime (IAOX) pathway. The pathway via IAM or IPA was the major route (s) to the biosynthesis of IAA in plants [43]. The expressions of auxin biosynthesis genes *OsYUCCA1* and *OsYUCCA6* were significantly downregulated, but the expressions of auxin metabolism genes *OsGH3.2* and *OsGH3*.8 were markedly increased in rice black-streaked dwarf virus (RBSDV)-infected rice plants. Meanwhile, IAA concentrations were significantly lower in the RBSDV-infected plants than those in the CK-inoculated controls [12]. In our study, the key genes expressions of the IPA pathway (tryptophan-IPA-IAD-IAA), including *TAA1*, *TAR2*, *YUCs,* and *ALDHs,* were decreased significantly in PMV-infected plants (Figure 8 and Table S16), suggesting that IPA pathway might be the main route for IAA biosynthesis in pecan and was restrained by PMV. Furthermore, numerous of the auxin-signaling IAA genes were also downregulated except for CIL1293S0053 (Table S15). However, consistent with the expression of *GH3* in rice against RBSDV, the expression of *GH3.1s* (CIL0330S0014 and CIL1456S0019) was upregulated in PMV-infected plants, especially in the yellow leaves (Table S15). Previous reports showed that the *OsGH3* family genes reduced auxin content by catalyzing the conjugation of IAA to amino acids [12]. These results showed that PMV infection might downregulate the auxin content.

In our study, the expressions of JA biosynthesis-related genes (*LOX2.1s*, *OPR1*, *OPCL1*, *ACX2*, *PED1*) and JA signaling-related genes (*JAZs* and *MYC2*) were markedly increased in PMV-infected plants compared to the non-infection plants (Figure 9, Tables S15 and S17). Previous reports showed that the expressions of JA biosynthesis-related genes (*OsOPR7*, *OsLOXs*) and JA signaling-related genes (*OsMYC2*, *OsJAZ12*) were markedly increased in RBSDV-infected Nip plants compared to mock-inoculated Nip plants, and the JA concentration was more than six-fold greater in RBSDV-infected Nip than that in mock-inoculated Nip plants [12]. These results showed JA biosynthesis played a positive role against pathogen attacks.

Auxin signaling was required to activate the JA pathway upon viral infection, and auxin positively interacted with the JA pathway against necrotrophic pathogens [12]. It was very interesting that almost all auxin-signaling IAA gene transcripts were downregulated in PMV-infected plants, but the expression of CIL1293S0053 was upregulated. The expression of *PR1* (CIL0232S0001) was remarkably enhanced in PMV-infected plants compared to CK. However, the expression levels of SA signaling genes (*NPR5*, *TGA7*) were downregulated (Table S15). Therefore, the expressions of auxin and JA biosynthesis and metabolism genes of PMV infection in pecan plants had similar results with those of RBSDV-infected rice plants, deducing that disruption of auxin signaling affected the activation of the JA pathway, but not the SA pathway, in response to PMV infection in pecans.

### 3.5. Cytokinins Signaling Difference Was One of the Key Reasons for Leaves Color in PMV Infection Pecan Plants

Cytokinins play an important role in the modulation of plant innate immunity. Cytokinins regulated the host defense responses either positively or negatively depending on the concentrations of cytokinins available at the infection site [11,44]. Isopentenyltransferase (IPT) was the key enzyme for the biosynthesis of cytokinins, and it catalyzed the rate-limiting step in cytokinin biosynthesis [45,46]. Transgenic tobacco plants’ overexpression of *IPT* gene overproduced cytokinin and resistance to tobacco necrosis virus (TNV). The number of virus-induced necrotic lesions was significantly lower in transgenic tobacco than those in WT [47]. Infection of barley plants by *Pyrenophora teres* and maize plants by *Drechslera maydis* were characterized by “green island” formation and high accumulation of cytokinins at infection sites [10]. The expression of the *IPT* gene (CIL1573S0016) was downregulated significantly in yellow leaves but upregulated significantly in green leaves (G2 and G3 samples) of PMV-infected pecan plants in our study (Figure 10 and Table S8). Correspondingly, three *AHP4* genes had the same expression patterns (Figure 8 and Table S15). These results showed that cytokinin biosynthesis and signaling transductions were enhanced in green leaves but restrained in yellow leaves of PMV-infected pecan plants, indicating cytokinins might be involved in triggering defense responses against PMV in green leaves. Cytokinins induced resistance independently [45] or dependently [48] of the SA signaling system. Cytokinins upregulated plant immunity via an elevation of SA-dependent defense responses. The crosstalk between cytokinin and SA signaling networks might help plants fine-tune defense responses against pathogens [11]. For SA biosynthesis, isochorismate synthase (ICS) and phenylalanine ammonia-lyase (PAL) were key enzymes in the isochorismate pathway and phenylpropanoid pathway, respectively, originated from chorismite [49]. The expression of *ICS* (CIL1563S0001) and *PAL* (CIL1348S0047) genes were both downregulated significantly in all samples in PMV-infected pecan plants, while the expression levels of *PAL* in green leaves were higher than those in yellow leaves (Table S8). Similarly, the *NPR5* (CIL0128S0002) and *TGA7* (CIL1564S0058) genes had the same expression patterns as *PAL*. Moreover, the *PR1* (CIL0232S0001) gene transcript was increased remarkably in all samples, and the expression level of *PR1* in green leaves was higher than those in yellow leaves. Collectively all the expressions differences of *IPT*, *AHP*, *PAL*, *ICS*, *NPR5*, *TGA7,* and *PR1* in green and yellow leaves of PMV-infected pecan plant, we deduced that (1) SA biosynthesis was inhibited by PMV, (2) the enhancement of cytokinins biosynthesis and signaling transductions resulting in cytokinin accumulation in green leaves was highly effective in activating plant immune responses, (3) cytokinins accumulation in green leaves induced the partially SA biosynthesis and gained comparatively higher SAR compared to yellow leaves. Cytokinins signaling in concert with SA signaling activated defense responses against pathogen infection [11]. Taken together, cytokinins signaling difference was one of the key reasons for leaf color in PMV-infected pecan plants.

### 3.6. Carbohydrate Partitioning Was Dramatic Changed in PMV Infection Pecan Plants

Pathogens attack significantly altered carbohydrate metabolism [6,50]. The effect on sugar levels varied considerably between different plant–pathogen interactions. Starch levels were significantly lower in the cucumber mosaic virus-infected melon plants [51] but higher in the sugarcane yellow leaf virus (SCYLV) infected sugarcane [52], tomato treatment with *Botrytis cinerea* [53] and grapevine-infected *Plasmopara viticola* [54]. In our study, the expressions of starch biosynthesis genes encoding granule-bound starch synthase 1 (WAXY) and glucose-1-phosphate adenylyltransferase (GLGC) were downregulated significantly in yellow leaves of PMV-infected pecan plants, and the expressions level of these genes in yellow leaves were lower than those in the green level of PMV-infected pecan plants. However, the starch-degrading enzyme genes *alpha-amylase 1* (*AMY1s*) were upregulated significantly in the Y3 sample. Previous reports showed that WAXY was responsible for amylose synthesis [55], and AMY1 was shown to have a significant function in leaf starch degradation in rice [56]. The expression of the *AMY1* was induced in grapevine treatment with *P*. *viticola* [54]. These results suggested that starch content might be decreased due to biosynthesis reducing and degradation increasing in PMV-infected pecan plants.

Soluble sugar content was increased upon pathogen attack in plants [51,57,58]. Both glucose and fructose levels were higher in grapevine leaves of *P. viticola* infected plant than those in control leaves [54]. Sucrose was the main form of sugar transported in plants, and invertase (beta-fructofuranosidfructohydrolase) was the enzyme that was capable of breaking down α-1,4-glycosidic linkage between D-glucose and D-fructose of sucrose. Invertase had various isoforms with distinct characteristics [59]. In our study, PMV infection induced the cell wall *INV* gene (CIL1317S0076) expression, suppresses the acid *INV* gene (CIL1264S0043) expression, alters the insoluble *CWINV1* (CIL1506S0011) expression (Table S19). Enhanced expression and activity of cell wall invertases have been reported in several plant–pathogen interactions [57]. Albugo candida induced the expression of cell wall invertase 1 in *Arabidopsis* [60]. Moreover, the cell wall *INV* (CIL1317S0076) transcript was upregulated significantly in yellow leaves compared to green leaves of PMV-infected pecan plants (Table S19).

The expressions of starch biosynthesis genes (*GLGCs* and *WAXYs*) were downregulated strikingly, but the expressions of starch degradation genes (*AMY1s*) were upregulated remarkably in Y3 samples compared to CK in our study (Table S19), suggesting that starch content might be decreased. The cell wall *INV* (CIL1317S0076) transcript was increased startlingly in Y3 samples. The inhibition of starch accumulation was probably due to the increased demand for soluble sugars required to maintain the high respiration rate [51]. Correspondingly, the photosynthesis and photosynthesis-antenna proteins-related gene expressions were downregulated in the Y3 sample of PMV-infected plants (Figure 7 and Table S14). Therefore, the repression of genes encoding photosynthesis and photosynthesis-antenna proteins-related might also explain the reduction in the photosynthetic efficiency. A lower rate of photosynthesis was associated with an increase in invertase activity in our conditions. These results showed the repression of photosynthesis and the induction of sink metabolism in the infected tissue led to dramatic changes in carbohydrate partitioning.

### 3.7. Disturbance of the Ribosome Biogenesis Might Enhance the Resistance to PMV Infection and Lead to Leaves Staying Green in Pecans

Ribosomes were universally important in biology, and their productions were dysregulated by developmental disorders, cancer, and virus infection [61,62]. The mature 80S ribosome in the cytoplasm comprised the 40S small subunit and the 60S large subunit in eukaryotes. 40S ribosome subunits contained ~30 ribosomal proteins of the small subunit (RPSs), and 60S ribosome subunits contained ~40 to 48 ribosomal proteins of the large subunit (RPLs) [63,64]. Ribosome biogenesis was a complex process that involved transcription of the ribosomal DNA (rDNA), precursor-rRNA (pre-rRNA) processing, RNA modifications, as well as assembly of the rRNAs with ribosomal proteins (RPs), and assembly factors [65,66].

Cai et al. demonstrated that Runx1 loss decreased ribosome biogenesis and translation in hematopoietic stem and progenitor cells and conferred resistance to endogenous and genotoxic stress [67]. Proteome comparison and host non-homology analysis resulted in 3605 pathogen-specific conserved core proteins in response to Leishmaniasis. Eight hub proteins were identified through protein-protein interaction network analysis, including ribosomal proteins S17 (LBRM2903_34004790) and L2, U3 small nucleolar RNA-associated protein, and so on [68]. Proliferative diseases such as cancer were associated with hyperactivated rRNA synthesis and ribosome biogenesis. Ribosome biogenesis restricts innate immune responses to virus infection and DNA [61]. These results showed that disturbance of the ribosome biogenesis enhanced the resistance to virus infection. Meanwhile, ribosome biogenesis was quickly inhibited by low temperatures in rice, which shed light on the link between ribosome biogenesis and environmental acclimation in crop plants [69]. In our study, 89 DEGs related to the ribosome pathway (Ko03010) and 19 DEGs related to ribosome biogenesis in eukaryotes (KO03008) were detected in PMV-infected samples compared to CK (Tables S20 and S21). Moreover, plenty of these genes were downregulated significantly in green leaves (G2 and G3 samples) but were not obviously changed in PMV-infected yellow leaves. Ribosome biogenesis in vivo was highly energy consuming and strictly orchestrated by internal and external signals to meet the demand for mature ribosomes in mRNA translation [66]. Taken together, these results deduced that disturbance of the ribosome biogenesis might enhance the resistance to PMV infection in pecan and lead to leaves staying green. Specialized ribosomes have been suggested to hold precise functional roles, especially in the context of immunology and cancer. Ribosomes have also been implicated in the immunosurveillance of cancer and other types of pathogenic cells. More direct evidence has shown the association of RP gene mutations with numerous cancers, raising the prospect of the existence of oncoribosomes. Anecdotal evidence suggests that ribosome has been linked to human disease [62]. However, the mechanism of the ribosome in plant resistance to disease was not well studied. A detailed study will be needed for the regulation mechanism of ribosome resistance to PMV invasion in pecan.

### 3.8. Fatty Acids-Derived Signaling Was One of the Important Defense Pathways in Resistance to PMV Infection Pecan

Fatty acids were emerging as important molecules that participated in diverse biological processes, including the regulation of different defense/cell death signaling pathways. In particular, de novo fatty acid biosynthesis was often required for numerous viruses’ replication in cellular membranes to generate new complex structures [70]. AMPK activity inhibited fatty acid synthesis while promoting fatty acid degradation [71]. AMPK directly phosphorylated acetyl-CoA carboxylase (ACC), the first rate-limiting enzyme in fatty acid synthesis, thereby inactivating the rate-limiting enzyme in the metabolism of fatty acids [72]. In particular, ACC catalyzed the irreversible conversion of acetyl-CoA to malonyl- CoA, a key metabolite that played multiple roles in fatty acid metabolism. Moser et al. reported that AMPK was activated during rift valley fever virus (RVFV) infection in humans, leading to the phosphorylation and inhibition of acetyl-CoA carboxylase, suggesting that AMPK potently inhibited fatty acid synthesis, restricted infection of the Bunyavirus [73]. Therefore, AMPK was an important component of the cell’s intrinsic immune response that restricted infection through a novel mechanism involving the inhibition of fatty acid metabolism [73]. In our study, the expression of *ACC* (CIL1396S0003) was downregulated in all infected pecan leaves (Table S18), showing that the fatty acid synthesis was inhibited and PMV infection was restricted.

Fatty acids play a critical role in maintaining membrane integrity due to their important energy storage molecules. In plants, fatty acid biosynthesis occurs exclusively in the plastids and requires the activity of the soluble stearoyl-acyl carrier protein-desaturase (SACPD). This enzyme introduced a *cis*-double bond at the carbon position 9 of the saturated fatty acids, stearic acid (18,0), to form the monounsaturated FA, oleic acid (C18,1). Upon its synthesis, C18,1 could enter glycerolipid synthesis via the prokaryotic pathway in the chloroplasts [74]. *Arabidopsis ssi2* mutation (encode stearoyl-acyl carrier protein-desaturase) plants reduced the level of C18,1, resulting in pronounced stunted phenotype and the appearance of visible cell death lesions on leaves, accumulated high levels of SA and overexpress *pathogenesis-related* (*PR*) genes, enhanced resistance to bacterial and oomycete pathogens [75]. Reducing the levels of C18,1 in the *Arabidopsis ssi2* mutation plants increased transcript levels of *R* genes and conferred resistance to turnip crinkle virus (TCV) [76]. Similarly, silencing of *GmSACPDs* showed severely stunted phenotype, altered leaf morphology, and the appearance of necrotic lesions on leaves in soybean, reduced C18,1, increased stearic acid, increased SA accumulation, and constitutively expressed *PR* genes and some *R* genes [77]. Cotton GhSSI2 isoforms, including GhSSI2-A, GhSSI2-B, and GhSSI2-C, played a dominant role in the cotton C18:1 pool. Suppressing the expression of *GhSSI2s* reduced the C18:1 level and enhanced cotton *Verticillium wilt* and *Fusarium wilt* resistance. Knockdown of *GhSSI2s* triggered a lesion mimic phenotype with an elevated SA level but without activating the JA signaling pathway [13]. Rice *OsSSI2*-knockdown plants showed markedly enhanced resistance to leaf diseases, such as the blast fungus *Magnaporthe grisea* and leaf blight bacterium *X*. *oryzae* pv. oryzae [78]. Thereby, C18,1 functioned as a signaling mediator in plant resistance against several pathogens [79]. In our study, the expressions of three *SACPD* genes (CIL0436S0014, CIL1297S0040, and CIL0373S0007) were downregulated in all PMV-infected plant leaves (Table S18); moreover, the *PR1* gene (CIL0232S0001) and numerous *R* genes were upregulated. These results suggest that the C18:1-regulated pathway may be specifically targeted during pathogen infection and that altering the C18:1 level might serve as a unique strategy for promoting disease resistance.

Generally, the cuticle includes a cutin polymer matrix (intracuticular wax) and cuticular wax (epicuticular wax) embedded within the cutin polymer. Cutin was made of saturated C-16-hydroxy and partially unsaturated C-18-hydroxy and C-18-epoxy fatty acids. In contrast, cuticular waxes were complex organic solvent-extractable mixtures of monomeric C-20 to C-60 aliphatics. Very-long-chain fatty acids (VLCFAs) were required in all plant cells for the synthesis of essential membrane lipids and also used as precursors of protective cuticular waxes and suberin in leaves, epidermis, and roots. They played crucial physiological and structural roles in plants’ response to abiotic and biotic environment stresses [80]. VLCFA biosynthesis begins with the elongation of saturated and monounsaturated C16 and C18 fatty acids produced in the plastid. Elongation of FAs occurs in the endoplasmic reticulum and involves four successive reactions catalyzed by four core enzymes perform, including 3-Ketoacyl-CoA synthase (KCS, CUT, FDH) [81], 3-Ketoacyl-CoA reductase (KCR), 3-Hydroxyacyl-CoA deshydratase (HCD), and enoyl-CoA reductase (ECR). The first step was the condensation of an acyl-CoA and a malonyl-CoA. This rate-limiting step was catalyzed by a condensing enzyme KCS. Heterologous expression of Newhall navel orange *CsKCS6* in *Arabidopsis* significantly increased the amount of VLCFAs in the cuticular wax on the stems and leaves. The transgenic lines experienced less water loss and ion leakage after dehydration stress and displayed increased survival under drought stress treatment compared to that of the wild-type (WT) plants [82]. Previous reports showed that mutation in KCS1 and KCS6 in barley and *Arabidopsis* had the glossy, wax-deficient phenotype, influencing the water barrier properties of the cuticle, which in turn affects the germination of barley powdery mildew fungus, suggesting that VLCFA-derived wax components were also shown to affect plant response to biotic stress [83,84].

Relation to the regulation of VLCFAs biosynthesis pathways has been reported previously. Overexpression of *AtMYB30* enhanced the expression of *KCS*, *FDH,* and *LACS* genes for VLCFA synthesis, promoted the accumulation of VLCFAs, and resulted in subtle changes in cuticle composition, while did not alter de novo fatty acids biosynthesis and PUFA, JA, and oxylipin accumulation [85]. Thus, AtMYB30 regulates the expression of FA elongase complex genes, involved in the generation of novel VLCFA-derived signals able to regulate the HR cell death and defense responses in *Arabidopsis* [85]. *MYB96* directly induced the expression of genes involved in cuticular wax biosynthesis, such as *AtKCS1*, *AtKCS2*, *AtKCS6,* and *AtKCR1* [86,87,88]. *CsMYB96* directly activated *CsKCS20* to promote the formation of C22 and C24 VLC acyl-CoAs and wax biosynthesis in citrus fruit [89]. Moreover, other MYB transcription factors, including MYB16 [90], MYB94 [91], and MYB106 [92], also participated in VLCFA and wax biosynthesis in many crops. In our study, plenty of MYB transcription factors were downregulated significantly in PMV-infected plant leaves compared to CK (Table S12). Moreover, fatty acid elongation metabolism genes, including *KCSs*, *FDH*, *CUTs*, *KCRs*, and *ECR,* were also downregulated significantly in PMV-infected plant leaves (Figure 11 and Table S18). Combined with the results already reported previously, these results suggested that MYB transcription factors regulated the expressions of *KCSs*, and biosynthesis of VLCFAs, involved in response to resistance to PMV infection in pecans.

To summarize, we show that PMV infection in pecans (1) inhibited the expression of first rate-limiting enzyme (ACC) in fatty acid synthesis, (2) downregulated the expression of *SACPDs*, could lead to reducing the level of C18:1, enhanced the expressions of *PR1* and amount of *R* genes, (3) reduced the expressions of numerous MYB transcription factors and key genes of VLCFA biosynthesis. These results could deduce that fatty acid synthesis was blocked, and oleic acid (C18:1) and VLCFA biosynthesis were also restrained in PMV-infected pecans. Consequently, fatty acids-derived signaling has also started to emerge as one of the important defense pathways, and the C18:1-regulated pathway might be specifically targeted during PMV infection in pecans. These findings broaden our knowledge regarding the lipid signal in disease resistance and provide novel insights into the molecular mechanism of PMV resistance.

## 4. Materials and Methods

### 4.1. Plant Materials and Growth

A naturally occurring leaf-variegated plant of pecan was obtained from millions of one-year-old pecan seedlings from Nanjing, Jiangsu province in China, in May 2019. The plant was grown at the Institute of Botany, Jiangsu Province, and the Chinese Academy of Sciences, China. The early one-year-old leaf-variegated plants bear yellow margins and green interior leaves (Mosaic leaf, ML, Figure 1B). When developing a pinnately compound leaf, the plant has half green leaves and half yellow leaves (Figure 1C), and a pinnately compound leaf had half small green leaves and half small yellow leaves (Figure 1D), the small leaf had half a piece of yellow (Figure 1E). In contrast, the typical normal seedling exhibits dark green-colored leaves (Figure 1A). In order to prove that the symptom was reproducible, we grafted leaf-variegated pecan plant branches onto normal pecan seedlings through spring branch grafting in 2022. The result showed that the new leaves from the same grafted branches showed the same symptom as the original leaf-variegated pecan plant (Figure S1). So, the mosaic leaves (ML) were harvested in June 2019, the green leaves (G1, G2, and G3) and yellow leaves (Y1, Y2, and Y3) of different PMV-infected plants, and the leaves of healthy plants were harvested in June 2020, and leaves with the same symptoms were mixed for follow-up experiments in our study.

### 4.2. Paraffin Section

Paraffin sections were performed according to previously reported by Li et al. [93] with toluidine blue staining. The healthy and PMV-infected plant leaves were put into 20 vol of FAA fixative. The paraffin sections were prepared by dehydration, wax immersion, embedding, section, baking, dying, and sealing. Neutral gum was used as a sealant. Laser scanning confocal microscopy (Zeiss LSM 900, LSM Bioanalytik GmbH, Magdeburg, Germany) was used to observe the differences between healthy and PMV-infected leaves.

### 4.3. Sequencing, Assembly, and Analysis of Small RNA Libraries

In order to make clear whether the mosaic leaves plant was caused by a virus infection, deep sequencing and assembly of virus-derived small interfering RNAs were performed according to the workflow of VirusDetect [14]. The mosaic leaves (ML) were harvested for small RNA sequencing and analysis. Total RNA was extracted using a Trizol reagent kit (Invitrogen, Carlsbad, CA, USA) following the manufacturer’s protocol. The total small RNAs ranging from 18 to 28 nucleotides (nt) were excised and ligated to adaptors. A small RNA library was constructed and sequenced using an Illumina HiSeq 2500 by Genedenovo Biotechnology Co., Ltd. (Guangzhou, China). After trimming the adaptor sequences and filtering for transfer and ribosomal RNAs, 16–28 nt short reads were collected and subsequently analyzed. Single-base resolution maps of all redundant sRNAs, along with the genomes, were created using bowtie tools and in-house Perl scripts [16]. Briefly, de novo assembly of the unmapped small RNA reads was assembled using the Velvet (version 1.2.10) (EMBL-European Bioinformatics Institute, Wellcome Trust Genome Campus, Hinxton, Cambridge, England) [17]. These assembled contigs were then annotated to host genome, NCBI non-redundant protein sequences (NCBI Nr), NCBI non-redundant nucleotide sequences (NCBI Nt), GenBank virus nucleotide reference sequences, GenBank virus protein reference sequences. Virus-related contigs annotation information contains virus nucleotide reference sequences annotated, or virus protein reference sequences annotated but not by host genome and virus nucleotide reference sequences annotated, and viruses only annotated by NCBI Nr/Nt. To evaluate the effectiveness of the virus, the sRNAs, which were not matched to the host genome, were compared to nucleic acid sequences found in the virus reference sequences nucleotide database by assembly contigs blast. The raw sequencing data generated from this study have been deposited in NCBI SRA (http://www.ncbi.nlm.nih.gov/sra, last accessed on 10 August 2022) under accession number PRJNA868138.

### 4.4. RNA Isolation and cDNA Library Preparation and Sequencing

Total RNA extraction, mRNA enrichment, and cDNA library conduction construction were performed according to the report of Zhang et al. [94]. Briefly, Total RNA was extracted from the leaves of infected with PMV and healthy plants using a Trizol reagent kit (Invitrogen, Carlsbad, CA, USA). mRNA was enriched by oligo (dT) beads. The enriched mRNA was then fragmented into short fragments and reverse transcribed into cDNA by using the NEBNext Ultra RNA Library Prep Kit for Illumina (NEB#E7530, New England Biolabs, Ipswich, MA, USA). The purified double-stranded cDNA fragments were end repaired, poly(A) was added, and they were ligated to Illumina sequencing adapters. The resulting cDNA library was sequenced using an Illumina HiSeq 2500 by Genedenovo Biotechnology Co., Ltd. (Guangzhou, China). The raw sequencing data generated from this study have been deposited in NCBI SRA (http://www.ncbi.nlm.nih.gov/sra, last accessed on 9 August 2022) under accession number PRJNA867660.

### 4.5. Analysis of Differential Gene Expression

The raw reads, including adapters or low-quality bases, were filtered using fastp (v.18) to obtain the clean reads [95]. To identify and remove residual rRNA reads, the clean reads were then mapped to a ribosome RNA database using the Bowtie2 (v.2.28) short read alignment tool (Center for Bioinformatics and Computational Biology, Institute for Advanced Computer Studies, College Park, MD, USA) [96]. The remaining clean reads were mapped to the pecan genome (Cil.genome.fa, ftp://parrot.genomics.cn/gigadb/pub/10.5524/100001_101000/100571/, accessed on 1 November 2021) using HISAT2.2.4 (Center for Computational Biology, McKusick-Nathans Institute of Genetic Medicine, Baltimore, MD, USA) [97] with the parameter “–rna-strandness RF” and default settings for the other parameters. De novo assembly of the unmapped clean reads was assembled using the StringTie v.1.3.1 (Center for Computational Biology, College Park, MD, USA) [98], and the assembled sequences were regarded as novel genes. The raw read counts for each gene were calculated using HTSeq (v.0.6.0) (European Molecular Biology Laboratory, Heidelberg, Germany) [99]. The gene expression was calculated and normalized to reads per kilobase per million mapped reads (RPKM) [100]. Analysis of gene expression differences between the two groups was performed by DESeq2 software (European Molecular Biology Laboratory, Heidelberg, Germany) [101]. Transcripts with a false discovery rate (FDR) <0.05 and absolute fold change >1 were defined as DEGs. Gene ontology (GO) enrichment (*p*-value < 0.05) was studied by running all DEGs through the GO database (http://www.geneontology.org/, accessed on 1 November 2021) to further classify the genes or their products into terms (molecular function, biological process, and cellular component) to understand their biological functions. Pathway projects were performed according to the KEGG pathway database for pathway enrichment analysis of DEGs.

### 4.6. Illumina RNA-Seq Results Validations by qRT-PCR

Ten key DEGs were selected for qRT-PCR analysis to validate the Illumina RNA-seq results. RNA was isolated from leaves according to the report of Zhang et al. [94]. First-strand cDNA synthesis was performed using the PrimeScript RT Reagent Kit with gDNA Eraser (Takara, Dalian, China) according to the manufacturer’s protocol. The primer sequences used were designed based on gene sequences and the Beacon designer software (Port St. Lucie, FL, USA), as shown in Table S22 in this study. The qRT-PCR was carried out on an Applied Biosystems 7300 Real-Time PCR System (Applied Biosystems, Waltham, MA, USA) using TaKaRa Company SYBR Premix Ex TaqTM II (Perfect Real-Time, TaKaRa, code: DRR041A, Dalian, China) as previously reported [102]. The CiActin gene was used as a positive internal control [103]. The relative levels of genes to control Actin mRNAs were analyzed using the 7300 system software and the 2^−DDCt^ method [104].

## 5. Conclusions

**Figure 14:**
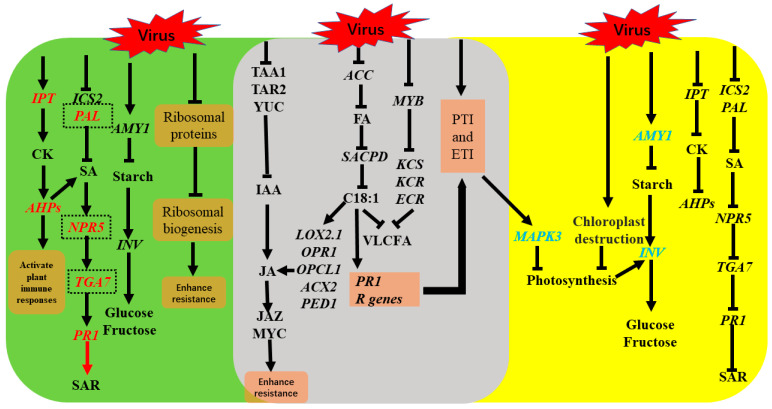
Proposed model for pecans: PMV interaction. Gray box shows common parts in green and yellow leaves infected with PMV. Green part shows the specific difference part in pecan infection PMV green leaves compared to pecan infection PMV yellow leaves or noninfected plants (CK) leaves. Yellow box shows the specific difference part in pecan infection PMV yellow leaves compared to pecan infection PMV green leaves or noninfected plant leaves (CK). The red text shows that the expressions of these genes in green leaves were upregulated and higher than those in yellow leaves of PMV-infected pecan plants. The blue text shows that the expressions of these genes in yellow leaves were upregulated and higher than those in green leaves of PMV-infected pecan plants. Red text with a frame shows that the expressions of these genes were suppressed, but the expression levels were higher in green leaves than those in yellow leaves of PMV-infected pecan plants. T shows that the gene transcripts were suppressed by PMV. Arrows show that the gene transcripts were induced by PMV.

In summary, we proposed a primary function network of pecan response to PMV-infected plants depending on our comparative transcriptome analyses of PMV infection in pecan and mixed with previous reports (Figure 14). PMV-infected pecan plants trigged PTI and ETI. Fatty acids-derived signaling pathway was one of the important defense pathways in resistance to PMV attack in pecan. Fatty acid C18:1 or its derivative generated within the chloroplasts was important in modulating signaling between SA- and JA-dependent defense pathways in *Arabidopsis* [105,106,107]. PMV infection in pecan reduced the level of oleic acid (C18:1) and enhanced the expressions of *PR1*. However, the key genes of SA biosynthesis (*PAL* and *ICS*) were downregulated. Meanwhile, disruption of auxin signaling affected the activation of the JA pathway. Thus, C18:1 and JA signals are involved in response to PMV infection in pecan. MYB TFs regulated the expressions of *KCSs*, and biosynthesis of VLCFAs, involved in response to PMV infection in pecan. We deduced that (1) cytokinin and SA synthesis were blocked, leading to plants losing immune responses and SAR, (2) cell and chloroplast structures were damaged, (3) activation of MPK3 inhibited photosynthesis, (4) the repression of photosynthesis and the induction of sink metabolism in the infected tissue led to dramatic changes in carbohydrate partitioning, which were the major reasons for leaf chlorosis in PMV-infected pecan plants. The green leaves of PMV-infected pecan plants had normal cell tissue structure and could establish a strong antiviral immunity system. Cytokinin biosynthesis and signaling transductions were remarkably strengthened, activating plant immune responses. Meanwhile, cytokinin accumulation in green leaves induced partial SA biosynthesis and gained comparatively higher SAR compared to those of yellow leaves. Disturbance of the ribosome biogenesis maybe enhance the resistance to PMV invasion in pecan and lead to leaves staying green. In future studies, we will validate the primary functional mechanism of pecan response to PMV infection through experimental methods, broadening our knowledge regarding the plant response to disease and providing novel insights into the molecular mechanism of PMV resistance.

## Data Availability

All relevant data are within the paper and its Supplementary Information Files, with the exception of the raw Illumina reads generated from RNA-seq experiments, which were deposited at NCBI Sequence Read Archive (PRJNA868138 and PRJNA867660).

## Supplementary Materials

The supporting information can be downloaded at: https://www.mdpi.com/article/10.3390/ijms232113576/s1.
