# Supplementary material for: Transcriptome Analysis Reveals a Comprehensive Virus Resistance Response Mechanism in Pecan Infected by a Novel Badnavirus Pecan Virus
Source: Int J Mol Sci. 2022 Nov 5;23(21):13576. doi: 10.3390/ijms232113576 (PMC9655656; doi:10.3390/ijms232113576)
Supplement: Supplementary file 1 [file ijms-23-13576-s001.zip › Figure S1,S2,S3,S4.pdf]

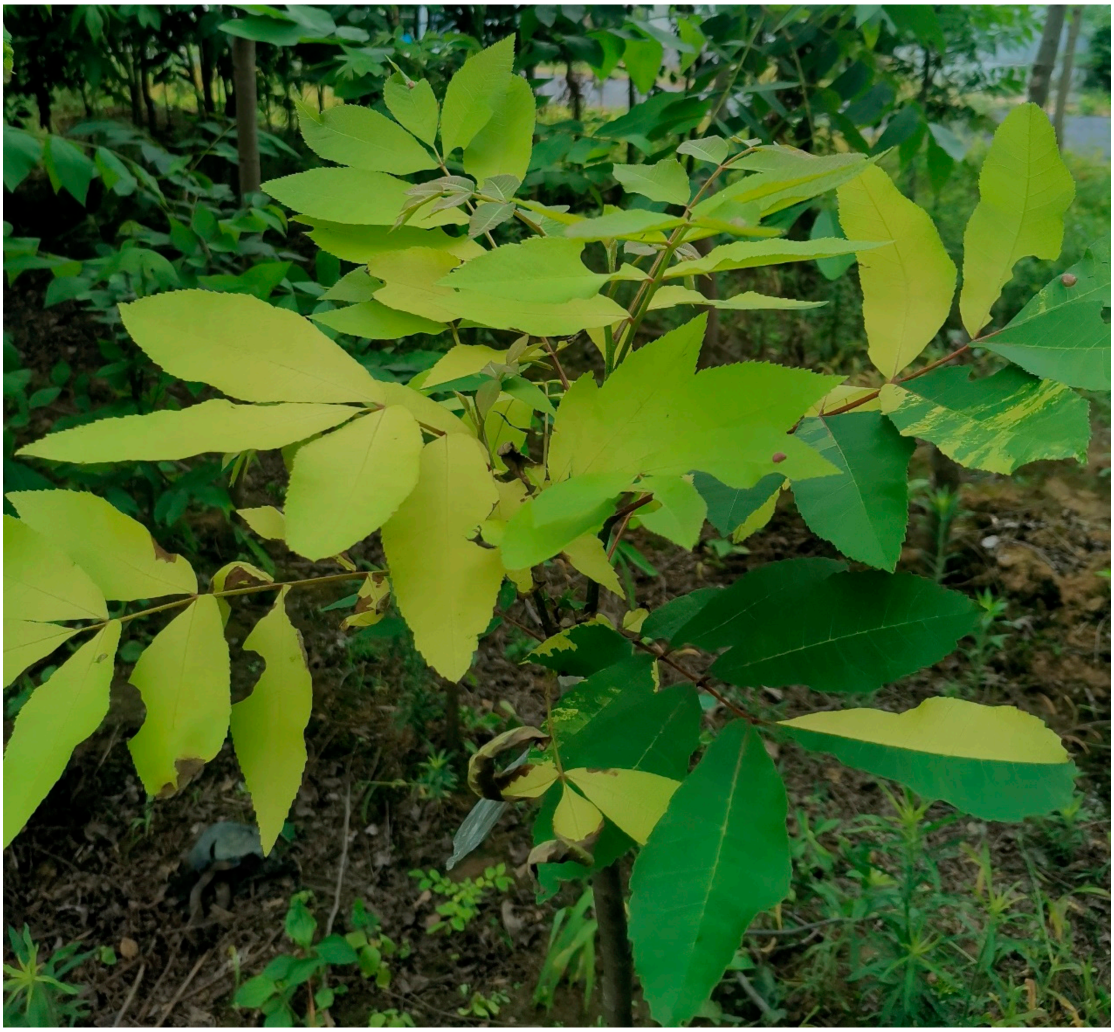

Figure S1. Phenotypic symptoms of grafted seedling

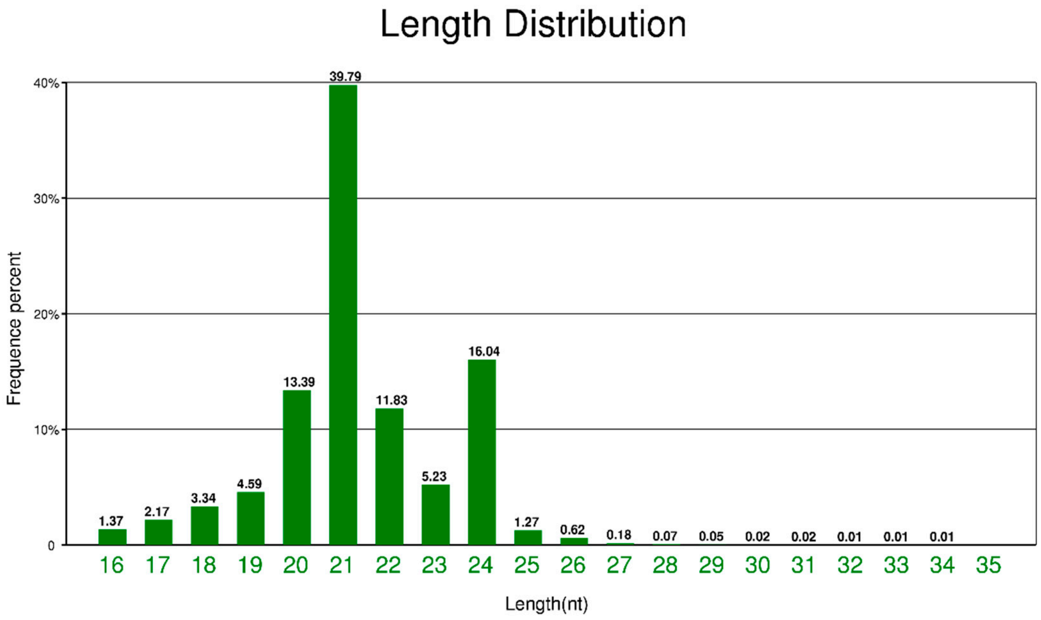

Figure S2. Size distribution of total sRNAs derived from PMV in pecan

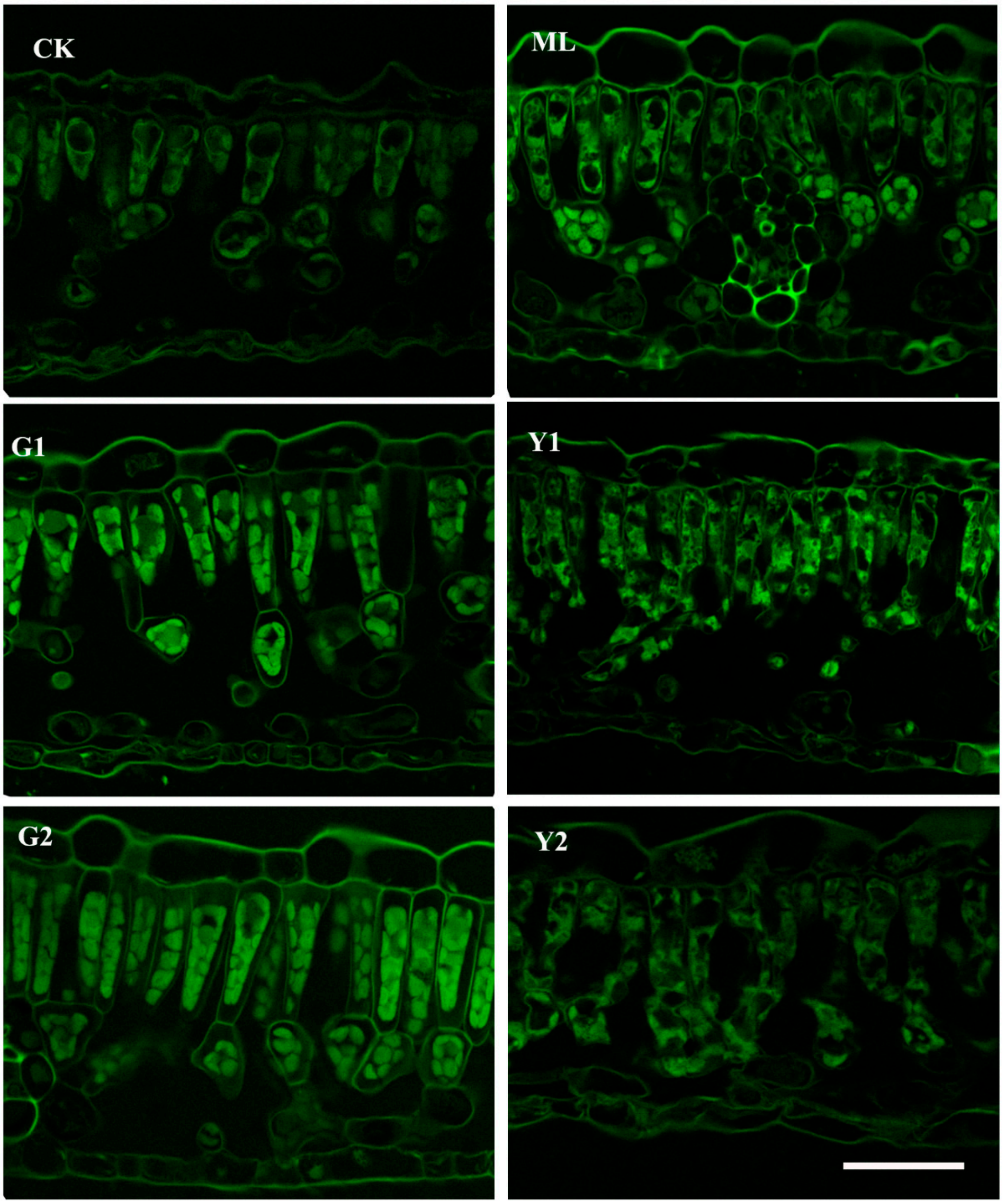

Figure S3. Leaf cross section structures analysis in healthy plant leaves (CK), the mosaic leaf (ML), the green leaves (G1 and G2) and yellow leaves (Y1 and Y2) of PMV infection plants. Bars=20 μm.

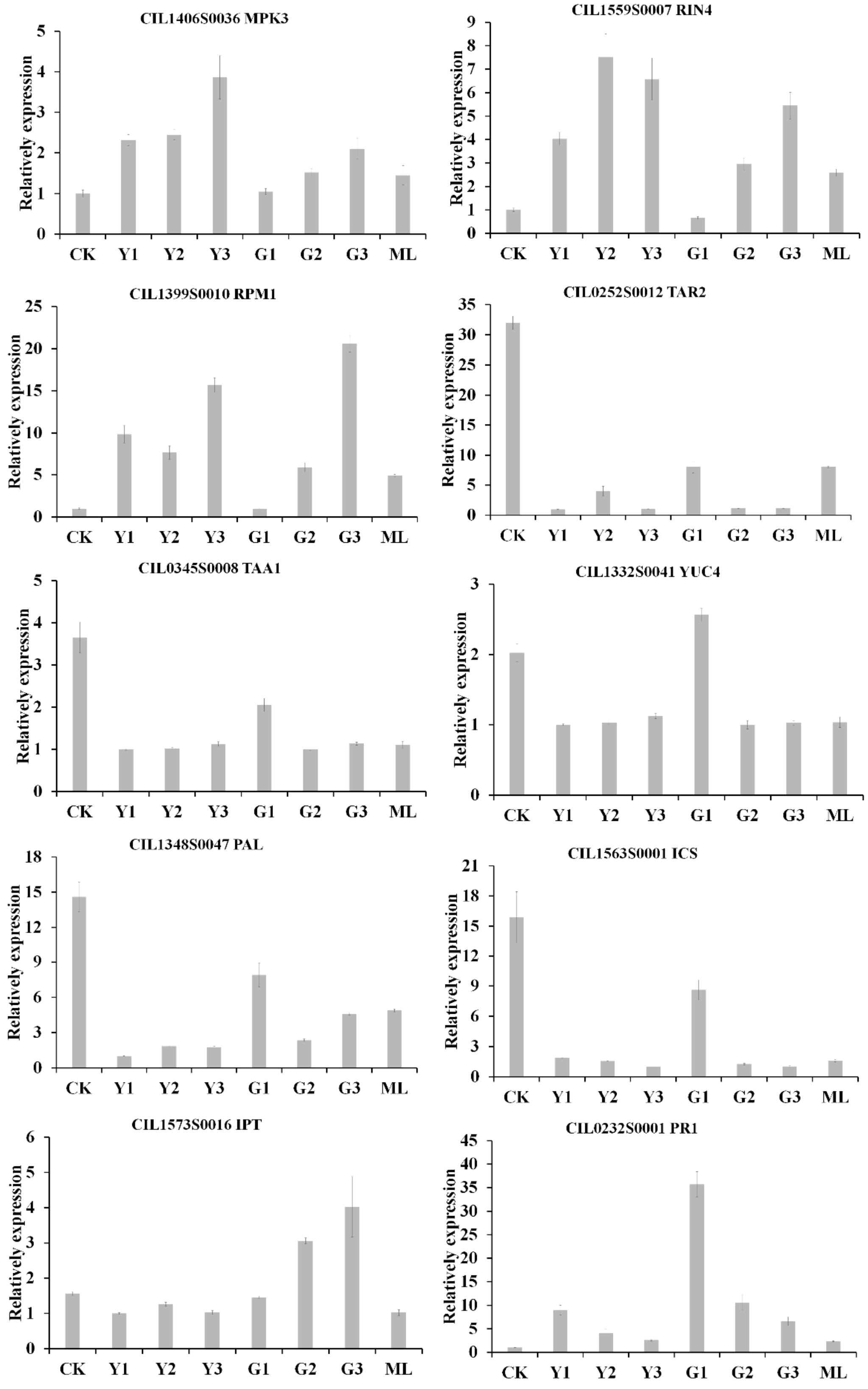

Figure S4. QRT-PCR analysis validation
